# n-Type organic semiconducting polymers: stability limitations, design considerations and applications

**DOI:** 10.1039/d1tc02048j

**Published:** 2021-06-15

**Authors:** Sophie Griggs, Adam Marks, Helen Bristow, Iain McCulloch

**Affiliations:** Department of Chemistry, Chemistry Research Laboratory, University of Oxford Oxford OX1 3TA UK sophie.griggs@chem.ox.ac.uk; King Abdullah University of Science and Technology (KAUST), KAUST Solar Center (KSC) Thuwal 23955-6900 Saudi Arabia

## Abstract

This review outlines the design strategies which aim to develop high performing n-type materials in the fields of organic thin film transistors (OTFT), organic electrochemical transistors (OECT) and organic thermoelectrics (OTE). Figures of merit for each application and the limitations in obtaining these are set out, and the challenges with achieving consistent and comparable measurements are addressed. We present a thorough discussion of the limitations of n-type materials, particularly their ambient operational instability, and suggest synthetic methods to overcome these. This instability originates from the oxidation of the negative polaron of the organic semiconductor (OSC) by water and oxygen, the potentials of which commonly fall within the electrochemical window of n-type OSCs, and consequently require a LUMO level deeper than ∼−4 eV for a material with ambient stability. Recent high performing n-type materials are detailed for each application and their design principles are discussed to explain how synthetic modifications can enhance performance. This can be achieved through a number of strategies, including utilising an electron deficient acceptor–acceptor backbone repeat unit motif, introducing electron-withdrawing groups or heteroatoms, rigidification and planarisation of the polymer backbone and through increasing the conjugation length. By studying the fundamental synthetic design principles which have been employed to date, this review highlights a path to the development of promising polymers for n-type OSC applications in the future.

## Abstract 2

This review summarises high performing n-type polymers for use in organic thin film transistors, organic electrochemical transistors and organic thermoelectric devices with a focus on stability issues arising in these electron transporting materials.

## Graphical abstract

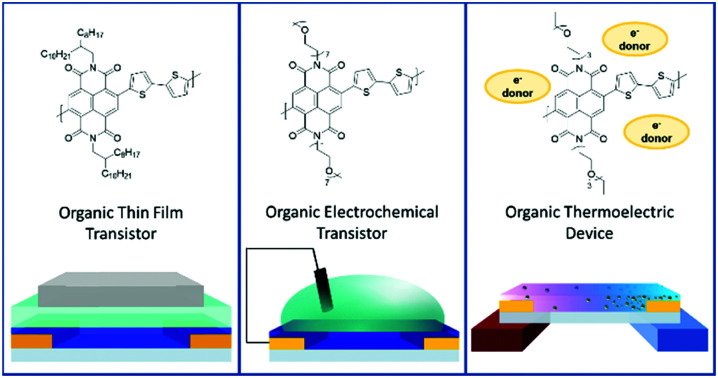

## 1. Introduction

### 1.1. Charge transport and morphology of n-type organic semiconductors

Organic semiconductors (OSCs) are organic materials that can readily switch from being a good conductor to a good insulator, whereby the process of doping or changes in the electric field control the state of the material.^1^ This usually involves the addition or removal of electrons to change the charge carrier density and allow the material to conduct a charge, with electron transporting polymers classed as n-type materials, whilst p-type materials transport holes.^2,3^ Where a material can transport both electrons and holes, it is referred to as ambipolar, and the vast majority of materials are able to exhibit some form of ambipolarity when the bias and electrodes are optimised.^4,5^ Chemical dopants can also be either n-type or p-type, depending on whether they are electron donors or acceptors, respectively. The addition of an n-type dopant donates electrons into the lowest unoccupied molecular orbital (LUMO) of a material, resulting in excess electrons to carry a charge. The energy of the LUMO of a material can be approximated as the electron affinity (EA), which is the energy released by adding an electron from vacuum energy to the innermost unfilled electron shell.^6^

Herein, this review will focus on electron transporting OSC polymers, and the considerations necessary for designing materials suited to the desired application. The prototypical n-type device for OSCs is the organic thin film transistor (OTFT), so the rationale behind the design of OSCs for n-type OTFTs is first discussed. A clear understanding of the features and properties of these structural motifs enables analysis of their further adaptation for application in the emerging n-type OSC based technologies, namely organic electrochemical transistors (OECT) and organic thermoelectric (OTE) generators. Current literature on polymeric OSCs is dominated by the high performance of p-type materials, whilst the development of electron transporting materials has consistently lagged. Improvements in n-type materials for OTFTs and OECTs are necessary to allow for the creation of complementary logic circuits, built with well-matched p-type materials, which lower the static power consumption, enabling faster circuit speeds, more complex circuits and increased operational stability.^7–9^ OTE generators also require OSCs for both p-type and n-type operation with well-matched electrical and thermal conductivities. To discuss the design of materials, first we must address the relationship between conjugation, charge transport and morphology, alongside the fundamental challenges with n-type materials and why high performing n-type materials are relatively scarce.^10^ It should be noted that theoretically the transport mechanisms utilised by electrons and holes are identical, so are not the limiting factor in the inferior performance of n-type materials.^11^

**Fig. 1:**
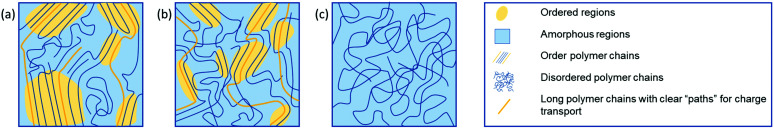
Graphic representation of the microstructure of a variety of polymeric materials. (a) Semi-crystalline polymer, (b) partial order due to short-range aggregates and (c) an amorphous structure. The yellow shading indicates ordered regions, and long polymer chains, indicated in yellow, represent clear “paths” for charge transport, significantly improving charge transport. Figure adapted from literature.^23^

The efficiency of charge transport in polymeric OSCs is governed by a number of factors both intrinsic to the polymer and dependent on device properties. Factors relating to the chemical structure of the polymer include reorganisation energy and transfer integrals, which in turn are influenced by the frontier molecular orbital energies and distributions, as well as the polymer conformation, molecular weight and sidechain composition.^12–15^ Device dependant factors include thin film morphology, charge carrier density, charge injection barriers and charge trapping.^16–18^ For example, the molecular weight of polymeric OSC materials can have a significant impact on the mobility of a material.^19^ This can be explained by noting that longer polymer chains act as connectors between crystalline regions within the microstructure (Fig. 1(a)). Without these, clear paths between ordered domains are limited and mobility is greatly reduced. It has been proposed, however, that for electron transporting polymers utilising a naphthalenediimide (NDI) backbone, the molecular weight is a less important factor for two reasons, the first being that the NDI unit can undergo a two-fold reduction to the dianion, thus charge remains localised on the NDI unit.^20^ The second reason is the degree of aggregation observed in solution, which reduces for lower molecular weight samples, resulting in more ordered backbones and larger regions of crystallinity.^21^ This second point also poses the question of the best methods for establishing the molecular weight of polymers, particularly where a material exhibits significant aggregation in solution. In these cases, the common method of using size exclusion chromatography, which includes gel permeation chromatography (GPC), may not be sufficient, and alternative methods, such as end group NMR spectroscopy analysis, to identify the absolute number of protons and determine the molecular weight may be required.^22^

A higher mobility is often recorded when defects and impurities in the material are minimised, thereby reducing the number of trapping sites and subsequently decreasing the number of immobile charge carriers. OSC polymers are most commonly semi-crystalline, with regions of crystallinity, dispersed in amorphous regions (Fig. 1). These regions of crystallinity can be categorised more specifically into areas of long- and short-range order.^23^ Charge transport occurs throughout the highly ordered regions as far as possible to avoid the large energy barrier associated with charges moving from ordered to amorphous regions. Unfortunately, transport through disordered domains is unavoidable and is often the bottleneck for charge transport in OSC polymers. Where charge transport must occur through amorphous regions, it is possible to assist this by ensuring polymer chains are of sufficient length to act as tie chains between regions of crystallinity.^24–26^

As such, materials that efficiently transport charge and are deemed “high mobility” generally contain adequate regions of high order, with short connecting sections of amorphous material.^27^ An example of a high-mobility n-type polymer is the branched NDI derivate **P(NDI2OD-T2)**, where large regions of crystallinity have been observed.^28^ This polymer also takes advantage of a face-on packing texture,^29^ with the aromatic polymer backbones stacked directly on top of each other, parallel to the substrate. This facilitates the hopping mechanism of electrons between chains, due to a stronger orbital overlap and interchain interaction.

In summary, more efficient packing leads to improved charge carrier transport, higher mobilities^29^ and short contact distances.^30–33^ Factors which alter the packing of a material are strong dipole–dipole interactions,^34,35^ degree of backbone planarity and steric locking of polymer backbones.^36,37^

### 1.2. Assessing the charge transport properties of n-type organic semiconductors

Typically, an assessment of charge transport properties is used to compare the performance of newly designed OSCs. Despite charge transport metrics being considered intrinsic properties of OSCs, in reality, the OSC electrical characteristics from which these transport properties are determined are dependent on the measurement technique and thin-film device type used to obtain them. New polymers are compared using mobility (OTFTs and OECTs), transconductance (OECTs) and conductivity (OTEs) values. These transport measurements are carried out in different device architectures; with either vertical or lateral transport; operating at varied applied biases; with significantly different charge carrier densities present; and in the case of OECTs and OTEs, with other charged components present in the films.^38–40^ The theoretical models applied to extract these transport properties were originally employed for ordered, defect and trap-free semiconducting materials, which OSCs are not.^41–43^ Even when using the same technique, decoupling the material properties of n-type OSCs from the methods used to determine them, so that design trends for the materials themselves can be established, is difficult. These methods include the choice of other materials used in the device, such as metal contacts, the processing of the OSC and the channel dimensions or volume over which the transport property is measured.

### 1.3. Stability of n-type organic semiconducting materials

**Fig. 2:**
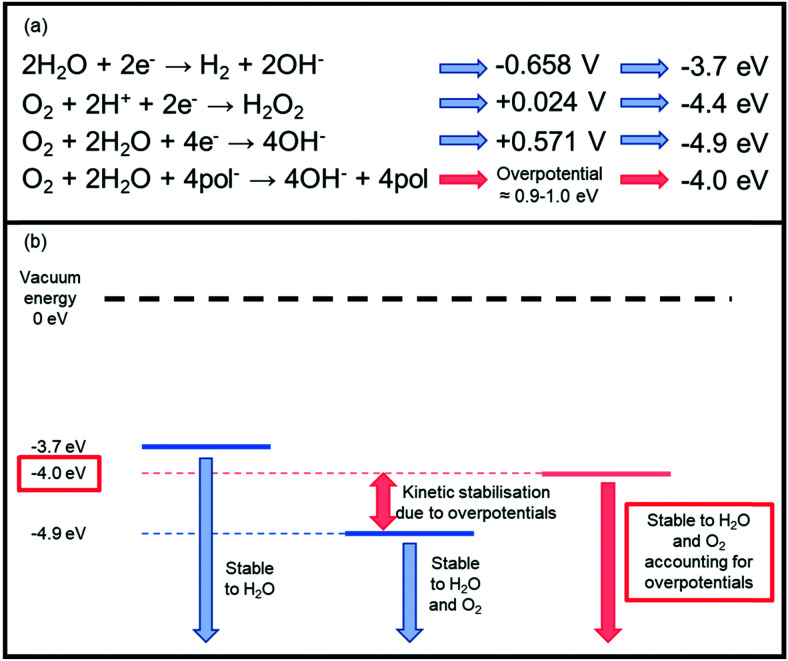
(a) The key reduction equations that cause inherent n-type thermodynamic instability and their associated energies. The redox potentials are measured *versus* the standard calomel electrode (SCE) at pH = 7.^47^ The corresponding LUMO levels have then been approximated from these redox potentials using the equation *E*_LUMO_ = *E*_red_ + 4.4 V.^47^ pol^−^ represents the anionic polymer species undergoing the redox reaction, which has an associated overpotential. (b) A schematic representation of the stability requirement of the LUMO energy level of an n-type material. This takes into account an overpotential of 0.9–1.0 eV, which is associated with the energetic barriers originating from penetration of the ambient species into the semiconducting material.^50^ Stability can generally be improved *via* two methods: either by operating devices under inert conditions or by designing materials with deeper LUMO levels.

There are three key types of instability which must be carefully managed to achieve high performing n-type materials, namely chemical, photochemical and electrochemical. Air sensitivity is a particular problem for the development of n-type OSCs.^44^ Rather than a chemical instability, these problems are associated with moisture and oxygen generated electron traps which impede n-type OTFT performance.^45^ Thermodynamic stability to redox reactions involving these ambient species is dictated by the energy of the LUMO level, whereby a shallow n-doped LUMO is at risk of oxidation by oxygen or water,^46^ as the reduction potentials of these species reside within the electrochemical window of most common n-type OSCs (Fig. 2).^47^

More specifically, water is reduced at potentials lower than −0.658 V (−3.7 eV), and oxygen can undergo reduction to hydrogen peroxide by electron transfer from the excited OSC negative polaron to a dioxygen molecule at +0.024 V (−4.4 eV).^47,48^ Oxygen can also undergo a four electron reduction at +0.571 V (−4.9 eV), so without taking overpotentials into account, for an n-type polymer to be stable to water, the doped (reduced) polymer should be oxidised at a potential higher than −0.658 V, furthermore for it to be stable to both oxygen and water, it needs to be oxidised higher than +0.571 V (Fig. 2).^47^ This initially appears unachievable, however, it has been empirically observed that a LUMO level below ∼−4 eV is necessary to ensure stability in ambient conditions.^49^ This can be explained through the concept of overpotentials, which is a free energy of activation that is required for the reaction to proceed, thus an excess voltage compared to the theoretically derived number is required to provide this free energy.^47^ In other words, although the reduction is thermodynamically favourable, it is kinetically hindered. This has been exemplified in studies investigating the stability of OTFTs based on OSCs with progressively deeper LUMO levels, from which an overpotential of around 0.9–1.0 eV was determined, which corresponds to a LUMO of −4 eV.^49^ The magnitude of the excess voltage will also be dependent on the OSC and the device configuration in which it is employed. Bearing this in mind as a design strategy moving forward is crucial if any attempts are to be made at creating thermodynamically stable n-type OSCs.

Oxygen can also be a threat to the stability of an OSC through the generation of singlet oxygen (^1^O_2_), which is formed by an energy transfer from the excited OSC triplet state, arising from intersystem crossing from the excited singlet state, to the triplet ground state of oxygen (^3^O_2_). This ^1^O_2_ is then able to undergo a 1,4 Diels–Alder addition in thiophene containing polymers, which leads to photobleaching.^48^ The simple solution to this is to encapsulate the device, removing all light sources, however this type of degradation must be predicted and accounted for in order to prevent it. OSC stability can also be improved by considering the close packed distances between polymer chains. Minimising these, for example through crystallisation, can provide a kinetic barrier to the diffusion of oxygen into the film, thus improving operational stability.^8,36,51^

Aside from the stability issues, a generic explanation for poor performance of n-types can be understood by examining the delocalisation of the LUMO in n-type polymers compared to that of the highest occupied molecular orbital (HOMO) in p-type polymers. In high performing p-type OSCs, while the HOMO may be slightly more prominent on the electron rich moiety, the HOMO is generally delocalised across the polymer backbone.^52,53^ This is not the case in common n-type materials, where the LUMO is more typically highly localised on the electron deficient component, causing electrons to become confined to the acceptor motif and electron mobility to decrease.^40^

Trapping of electrons in n-type OSCs can also occur on account of the device architecture, for example when employing silicon oxide substrates for n-type OTFTs, it has been shown that passivating the pendant hydroxyl groups by using a buffer dielectric/surface passivation is particularly important to prevent charge trapping, especially for n-type materials with shallow LUMO levels.^4,40^

Herein, we will discuss some fundamental properties and generic underlying design principles for electron transporting OSC materials, including synthetic manipulations of the energetics and optoelectronics of these materials, as well as providing high performing examples from literature.

### 1.4. Organic semiconducting material design strategies

**Fig. 3:**
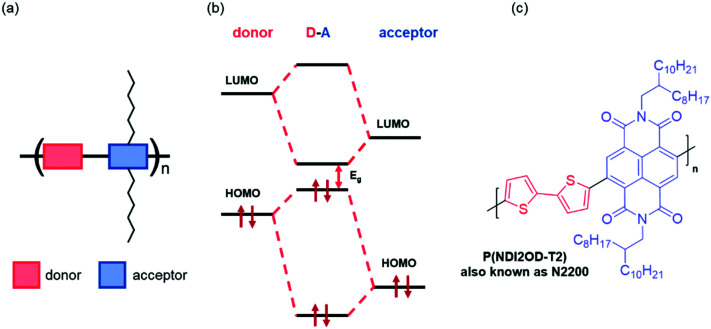
(a) Schematic of a donor–acceptor copolymer, (b) hybridisation of the molecular orbitals of the donor and acceptors monomers of a generic copolymer, and (c) an example of a donor–acceptor copolymer, *N*,*N*′-dialkylnaphthalenedicarboximide-dithiophene (**NDI2OD-T2**).^28^

The energy levels of OSCs can be tuned at the molecular level by chemical design. Most common types of OSC polymers consist of an alternating electron rich “donor” and an electron deficient “acceptor” component, namely donor–acceptor (D–A) polymers (Fig. 3(a)).^54^ Perturbation theory dictates that the orbitals of donor–acceptor polymers hybridise by combining the HOMOs and the LUMOs of the constituent monomers, redistributing their energy levels, and in turn the energy of the occupying electrons (Fig. 3(b)). This enables synthetic control and optimisation of these energy levels with relative ease and specificity. More specifically, by modifying the acceptor component, the LUMO energy level can be tuned to suit the application, for example in OECTs, it is mandatory that the LUMO is deep enough to support stability in ambient conditions (∼−4 eV), whilst in OTFTs, the requirement of a LUMO level deeper than −4 eV is not a limiting factor in the operation of the device. Broadly speaking, for n-type materials, these alterations to the acceptor motif aim to deepen the LUMO level, thus increasing the ambient stability of the material and facilitating electron injection.

One of the main synthetic reasons for D–A copolymers being so readily available for organic device applications is due to transition metal mediated coupling steps, which are facilitated by one of the two monomers having an electron rich conjugated system. The first high performing n-type material, poly{[*N*,*N*′-bis(2-octyldodecyl)-naphthalene-1,4,5,8-bis(dicarboximide)-2,6-diyl]-*alt*-5,5′-(2,2′-bithiophene)} (**NDI2OD-T2**), also known as **N2200** (Fig. 3(c)), has such a D–A motif.^28^ This copolymer exhibits a relatively deep LUMO (approximated through a large EA value of 3.9 eV), facilitating electron injection and giving reasonable stability.^28^ More generally, n-type materials tend to comprise of a selection of electron deficient units, including naphthalenediimide (NDI),^55^ diketopyrrolopyrrole (DPP)^56^ or isoindigo (IIG).^57^

**Fig. 4:**
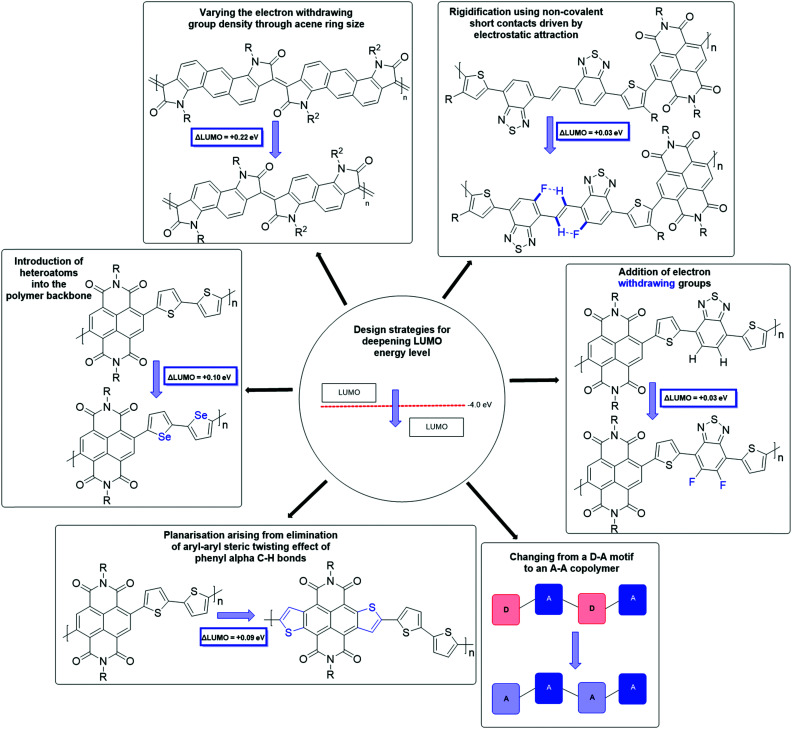
A summary of design strategies to deepen the LUMO level, which in turn improves n-type stability and performance.

The synthetic techniques for deepening the LUMO level of an OSC include extending the conjugation length,^58,59^ the introduction of heteroatoms into the polymer backbone,^60–62^ decreasing the dilution effect to reduce the electron density in the polymer backbone,^63^ increasing backbone planarization,^36,64,65^ addition of electron-withdrawing groups,^62,66,67^ and the use of an all acceptor motif to delocalise the LUMO (Fig. 4).^36,68^ These design strategies are explored in more detail in further material examples in the forthcoming sections.

Nitro, carboxyl, cyano and fluorine functionalities are examples of electron-withdrawing substituents through both inductive and resonance effects. These groups can also be combined with the use of alternating donor and acceptor monomers, to further reduce the electron density of the accepting component (Fig. 4).^54^ Alkyl sidechains are not only beneficial for promoting solubility of otherwise insoluble aromatic cores, but additionally have been shown to influence the molecular packing and charge transport properties of a material.^69^ This can either be beneficial or detrimental depending on the sidechain selection and their interaction, for example where a material exhibits interdigitation (*e.g.* as expected with PTEG-1^70^), long range order is achieved, and therefore mobility is increased.^71,72^ However, where branched chains are required to impart sufficient solubility, they can significantly impede charge transport.^73^ One solution is the introduction of a linear spacer between the backbone and the branching point, which maintains good close-contact distances and allows for solution-processability of the material.^74–76^ Other solubilising sidechains have also been explored in the field of OSCs,^77^ including those with ionic functionalities,^78^ hydrosilanes^79^ and most notably oligo(ethylene glycol) (OEG) chains,^80–82^ which will be discussed in detail in Section 3 as these are most applicable for use in OECT devices.

Herein, we set out three common device applications for n-type OSC materials and their requirements for high electron transport properties, namely organic thin film transistors (OTFT), organic electrochemical transistors (OECT) and organic thermoelectric generators (OTE).

## 2. n-Type organic thin film transistors

The synthetic design of polymeric OSCs for n-type OTFTs with the aim of achieving good environmental stability, operational stability and fast current modulation has been ongoing for over three decades.^83^ Towards this end, using the strategies discussed in Section 1.4, the LUMO, solubility, short-contacts between polymer chains and thin-film morphology of OSC materials can be controlled and manipulated. In this section, comparisons of the design strategies employed will be drawn between OSCs utilising the same backbone for which high electron mobilities are reported (mobilities > 0.1 cm^2^ V^−1^ s^−1^), where the electron mobilities dominate over any hole mobility also reported. The sections below set out the structures of polymers for discussion, along with some unclassified polymers, where it is not possible to draw direct comparisons to another material. These six main categories are naphthalenediimide (NDI) derivatives,^52,53,62,84^ naphthodithiophenediimide (NDTI) derivatives,^58,59^ polylactam/lactone derivatives,^60,85–87^ isoindigo (IIG) derivatives,^64,65^ diketopyrrolopyrrole (DPP) derivatives^88,89^ and acceptor–acceptor derivatives.^36^

**Fig. 5:**
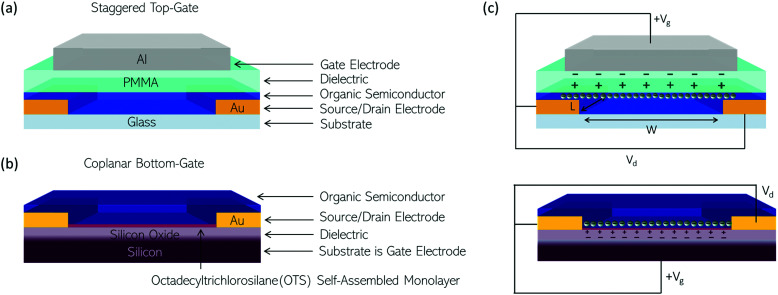
Illustrating two OTFT architectures used to test some of the polymers discussed in this review. (a) Staggered, top-gate with gold (Au) source and drain electrodes; a poly(methyl methacrylate) (PMMA) solution processed dielectric; and an aluminium (Al) gate electrode. (b) Coplanar, bottom-gate with a silicon substrate onto which a layer of silicon oxide is thermally grown as the dielectric; an OTS self-assembled monolayer used to passivate pendant hydroxy groups on the silicon oxide; gold (Au) source and drain electrodes. (c) When the OTFT is operated a drain voltage is applied across the OTFT channel with length (*L*) and width (*W*) defined by the source and drain electrodes. In an n-type OTFT a positive gate voltage (*V*_g_) is applied to the gate electrode polarising the dielectric and resulting in the accumulation of charge carriers in the organic semiconductor at its interface with the dielectric.

Typically, the performance of an OSC for OTFT applications is judged by the mobility determined directly from operating thin-film transistors. There is no standardised OTFT architecture for the testing of new OSC materials, as is clear from Tables 1–7. In an OTFT, current through the OSC is modulated by a gate electrode with a dielectric layer between it and the OSC. In practice, the materials used for this dielectric and its position relative to the source and drain electrodes (staggered *vs.* coplanar) and above or below the OSC layer (top-gate or bottom-gate) varies. Fig. 5(a) and (b) show examples of a typical staggered, top-gate and coplanar bottom-gate OTFT configuration, which are the predominant configurations used to test the OSCs discussed here. Contact engineering, choice of dielectric, OTFT configuration and device optimisation play a large role in dictating the electrical characteristics of an OTFT and thereby the mobility extracted.

**Table 1:** A selection of NDI derivative n-type OTFT materials with high reported performance (selection criteria of electron mobility greater than 0.1 cm^2^ V^−1^ s^−1^ and only including ambipolar materials where the n-type performance exceeds p-type), summarising their electron affinity (EA), weight and number average molecular weight (*M*_W_/*M*_n_), maximum reported electron mobility (*μ*_e_), ratio of on to off current (*I*_ON_/*I*_OFF_)^a^, threshold voltage (*V*_T_)^a^, OTFT channel length and width (*L*/*W*) used and summary of device structure. Where: coplanar (co.), staggered (st.), bottom gate (BG), top gate (TG); silicon oxide (SiO_2_), poly(methylmethacrylate) (PMMA), octadecyltrichlorosilane (OTS), pentafluorobenzenethiol (PFBT), octadecyltrimethoxysilane (OTMS); gold (Au), aluminium (Al), caesium carbonate (Cs_2_CO_3_); “—” represents the polymer layer

Polymer	EA^b^ (eV)	*M*_n_/*M*_w_ (kDa)	Max. *μ*_e_ (cm^2^ V^−1^ s^−1^)	*I*_ON_/*I*_OFF_	*V*_T_ (V)	Channel *L*/*W* (μm)	Device structure	Ref.
**P(NDI2OD-T2)**	3.91	26.6/85.1	6.40	10^7^	<10	20/2000	St.TG; Au – PMMA/Al	95
**PNDIF-T2**	4.01	28/57	3.93	10^5^	14	150/1500	St.BG; SiO_2_/OTS – Au	53
**P(NDI2HD-T2)**	—	97.8/244.5	1.90	>10^4^	35	50/1000	St.BG; SiO_2_/OTS – Au	96
**P(NDI2SiC6-T2)**	3.83	32/65	1.04	10^3^	22	—	St.TG; Au – PMMA/Al	101
**PNBSF**	3.88	56.1/238.2	3.50	—	48	5/1400	St.TG; SiO_2_/Au/OTS/PFBT – PMMA/Al	62
**PNBS**	3.81	39.7/147.6	0.29	>10^3^	42	5/1400	Co.BG; SiO_2_/Au/OTS/PFBT	62
**PNBTF**	3.85	48.5/221.6	2.20	>10^3^	60	5/1400	St.TG; SiO_2_/OTS/Au/PFBT – PMMA/Al	62
**PNBT**	3.77	36.7/148.6	3.20	>10^3^	38	5/1400	St.TG; SiO_2_/Au/OTS/PFBT – PMMA/Al	62
**PNDIF–TVT**	3.99	33/51	3.75	10^5^	15	150/1500	St.BG; SiO_2_/OTS – Au	53
**PNDI–TVT**	4.00	139/70	1.80	10^6^	13	10/1000	St.TG; Au/Cs_2_CO_3_ – PMMA/Al	52
**P3**	4.00	18.6/63.5	0.50	10^5^	15	50/500	St.TG; Au – PMMA/Au	102
**PNDIBS**	3.90	106.5/40.1	0.24	10^6^	12	100/1000	St.BG; SiO_2_/OTS – Au	103
**pSNT**	4.01	61.3/153.3	5.35	>10^6^	1	100/1000	St.TC; SiO_2_/OTMS – Au	104
**P4**	4.02	54.9/98.8	7.16	>10^6^	1	100/1000	St.TC; SiO_2_/OTMS – Au	105

a Where values aren’t reported directly in the text, these are inferred from given transfer plots.

b EA is an estimation of the LUMO, although neglects the electron binding energy.

**Table 2:** A selection of NDTI derivative n-type OTFT materials with high reported performance (selection criteria of electron mobility greater than 0.1 cm^2^ V^−1^ s^−1^ and only including ambipolar materials where the n-type performance exceeds p-type), summarising their electron affinity (EA), weight and number average molecular weight (*M*_W_/*M*_n_), maximum reported electron mobility (*μ*_e_), ratio of on to off current (*I*_ON_/*I*_OFF_)^a^, threshold voltage (*V*_T_)^a^, OTFT channel length and width (*L*/*W*) used and summary of device structure. Where: coplanar (co.), staggered (st.), bottom gate (BG), top gate (TG); silicon oxide (SiO_2_), octadecyltrichlorosilane (OTS), 3-[(*N*,*N*′-dimethylamino)propyl]triethoxysilane (MAPS); gold (Au); “–” represents the polymer layer

Polymer	EA^b^ (eV)	*M*_n_/*M*_w_ (KDa)	Max. *μ*_e_ (cm^2^ V^−1^ s^−1^)	*I*_ON_/*I*_OFF_	*V*_T_ (V)	Channel *L*/*W* (μm)	Device structure	Ref.
**PNDTI-BT-DT**	4.40	27.1/90.4	0.27	>10^2^	10	40/3000	St.BG; SiO_2_/OTS – Au	106
**PNDTI-BT-DP**	—	149.6/16147.0	0.24	>10^5^	13	40/1450	St.BG; SiO_2_/MAPS – Au	110
**PNDTI-BTT-DP**	4.40	20.5/51.9	0.31	>10^5^	4	40/1500	St.BG; SiO_2_/OTS – Au	58
**PNDTI-NTz**	4.20	15.7/27.2	0.21	>10^4^	15	40/1500	St.BG; SiO_2_/OTS – Au	59
**PDNTI-BTz**	4.10	14.4/42.2	0.10	10^4^	20	40/1500	St.BG; SiO_2_/OTS – Au	59

a Where values aren’t reported directly in the text, these are inferred from given transfer plots.

b EA is an estimation of the LUMO, although neglects the electron binding energy.

**Table 3:** A selection of polylactam/lactone n-type OTFT materials with high reported performance (selection criteria of electron mobility greater than 0.1 cm^2^ V^−1^ s^−1^ and only including ambipolar materials where the n-type performance exceeds p-type), summarising their electron affinity (EA), weight and number average molecular weight (*M*_W_/*M*_n_), maximum reported electron mobility (*μ*_e_), ratio of on to off current (*I*_ON_/*I*_OFF_)^a^, threshold voltage (*V*_T_)^a^, OTFT channel length and width (*L*/*W*) used and summary of device structure. Where: coplanar (co.), staggered (st.), bottom gate (BG), top gate (TG); silicon oxide (SiO_2_); CYTOP® fluoropolymer (CYTOP); gold (Au), aluminium (Al); “—” represents the polymer layer

Polymer	EA^b^ (eV)	*M*_n_/*M*_w_ (kDa)	Max. *μ*_e_ (cm^2^ V^−1^ s^−1^)	*I*_ON_/*I*_OFF_	*V*_T_ (V)	Channel *L*/*W* (μm)	Device structure	Ref.
**BDPPV**	4.24	37.6/89.4	1.10	>10^5^	5	10/200	St.TG; SiO_2_/Au – CYTOP/Al	87
**BDOPV-2T**	4.15	77.2/231.5	1.74	>10^4^	44	5/100	St.TG; SiO_2_/Au – CYTOP/Al	86
**AzaBDOPV-2T**	4.37	51.6/135.0	3.22	>10^4^	40	5/100	St.TG; Au – CYTOP/Al	60
**F4BDOPV-2T**	4.32	38.0/109.1	1.56	>10^3^	3	100/2000	St.TG; SiO_2_ – CYTOP/Al	85

a Where values aren’t reported directly in the text, these are inferred from given transfer plots.

b EA is an estimation of the LUMO, although neglects the electron binding energy.

**Table 4:** A selection of isoindigo (IIG) derivative n-type OTFT materials with high reported performance (selection criteria of electron mobility greater than 0.1 cm^2^ V^−1^ s^−1^ and only including ambipolar materials where the n-type performance exceeds p-type), summarising their electron affinity (EA), weight and number average molecular weight (*M*_W_/*M*_n_), maximum reported electron mobility (*μ*_e_), ratio of on to off current (*I*_ON_/*I*_OFF_)^a^, threshold voltage (*V*_T_)^a^, OTFT channel length and width (*L*/*W*) used and summary of device structure. Where: coplanar (co.), staggered (st.), bottom gate (BG), top gate (TG); silicon oxide (SiO_2_), poly(methylmethacrylate) (PMMA), octadecyltrichlorosilane (OTS); gold (Au), aluminium (Al); “—” represents the polymer layer

Polymer	EA^b^ (eV)	*M*_n_/*M*_w_ (kDa)	Max. *μ*_e_ (cm^2^ V^−1^ s^−1^)	*I*_ON_/*I*_OFF_	*V*_T_ (V)	Channel *L*/*W* (μm)	Device structure	Ref.
**PAIIDBT**	4.10	14.0/22.0	1.00	10^6^	30	20/1000	St.TG; Au – PMMA/Al	64
**PIIG-BT**	3.54	15.0/19.8	0.22	>10^7^	48	50/1000	St.BG; SiO_2_/OTS – Au	65
**P6F-C3**	3.80	52.9/81.5	4.97	>10^6^	55	80/5600	St.TG; SiO_2_/Au – PMMA/Au	115
**P6F-2TC3**	3.92	88.0/170.7	1.35	10^6^	45	80/5600	St.TG; SiO_2_/Au – PMMA/Au	115
**2FIID-BT2CN**	3.92	111.0/170.9	0.25	>10^4^	10	80/5600	St.TG; SiO_2_/Au – PMMA/Al	116

a Where values aren’t reported directly in the text, these are inferred from given transfer plots.

b EA is an estimation of the LUMO, although neglects the electron binding energy.

**Table 5:** A selection of diketopyrrolopyrrole (DPP) derivative n-type OTFT materials with high reported performance (selection criteria of electron mobility greater than 0.1 cm^2^ V^−1^ s^−1^ and only including ambipolar materials where the n-type performance exceeds p-type), summarising their electron affinity (EA), weight and number average molecular weight (*M*_W_/*M*_n_), maximum reported electron mobility (*μ*_e_), ratio of on to off current (*I*_ON_/*I*_OFF_)^a^, threshold voltage (*V*_T_)^a^, OTFT channel length and width (*L*/*W*) used and summary of device structure. where: coplanar (co.), staggered (st.), bottom gate (BG), top gate (TG); silicon oxide (SiO_2_), poly(methylmethacrylate) (PMMA), caesium fluoride (CsF), CYTOP® fluoropolymer (CYTOP); gold (Au), aluminium (Al); “—” represents the polymer layer

Polymer	EA^b^ (eV)	*M*_n_/*M*_w_ (kDa)	Max. *μ*_e_ (cm^2^ V^−1^ s^−1^)	*I*_ON_/*I*_OFF_	*V*_T_ (V)	Channel *L*/*W* (μm)	Device structure	Ref.
**BTI1-DPP**	3.34	24/—	0.27	>10^4^	16	5000/20 000	St.TGC; Au/CsF – CYTOP/Al	119
**BTI2-DPP**	3.42	23/—	0.48	>10^4^	27	5000/20 000	St.TG; Au/CsF – CYTOP/Al	119
**BTI3-DPP**	3.46	21/—	0.21	>10^5^	18	5000/20 000	St.TG; Au/CsF – CYTOP/Al	119
**PPyTDPP-TT**	3.75	120.0/352.0	0.48	>10^6^	64^a^	50/4500	St.TG; Au – PMMA/Al	120
**pTPDPP-TF**	4.10	24.4/125.3	0.10	10^4^	14	20/1000	St.TG; Au – PMMA/Al	88
**PPyDPP1-4FBT**	3.65	157.4/291.2	1.02	10^5^	28	80/5600	St.TG; SiO_2_/Au – PMMA/Al	121
**PPyDPP2-4FBT**	3.69	120.2/271.7	2.45	10^5^	25	80/5600	St.TG; SiO_2_/Au – PMMA/Al	121
**PPyDPP1-4FTVT**	3.66	102.7/181.8	1.19	10^6^	15	80/5600	St.TG; SiO_2_/Au – PMMA/Al	121
**PPyDPP2-4FTVT**	3.67	126.2/214.5	1.35	10^6^	21	80/5600	St.TG; SiO_2_/Au – PMMA/Al	121
**DPPTh-BT2CN**	3.67	155.0/207.7	0.35	>10^3^	15	80/5600	St.TG; SiO_2_/Au – PMMA/Al	116
**DPPPy-BT2CN**	3.75	275.0/357.5	0.30	>10^4^	1	80/5600	St.TG; SiO_2_/Au – PMMA/Al	116

a Where values aren’t reported directly in the text, these are inferred from given transfer plots.

b EA is an estimation of the LUMO, although neglects the electron binding energy.

**Table 6:** A selection of acceptor–acceptor derivative n-type OTFT materials with high reported performance (selection criteria of electron mobility greater than 0.1 cm^2^ V^−1^ s^−1^ and only including ambipolar materials where the n-type performance exceeds p-type), summarising their electron affinity (EA), weight and number average molecular weight (*M*_W_/*M*_n_), maximum reported electron mobility (*μ*_e_), ratio of on to off current (*I*_ON_/*I*_OFF_)^a^, threshold voltage (*V*_T_)^a^, OTFT channel length and width (*L*/*W*) used and summary of device structure. Where: coplanar (co.), staggered (st.), bottom gate (BG), top gate (TG); silicon oxide (SiO_2_), poly(methylmethacrylate) (PMMA), hexamethyldisilazane (HMDS), caesium fluoride (CsF), CYTOP® fluoropolymer (CYTOP); gold (Au), aluminium (Al); “—” represents the polymer layer

Polymer	EA^b^ (eV)	*M*_n_/*M*_w_ (kDa)	Max. *μ*_e_ (cm^2^ V^−1^ s^−1^)	*I*_ON_/*I*_OFF_	*V*_T_ (V)	Channel *L*/*W* (μm)	Device structure	Ref.
**P4**	4.20	134/538	0.2	>10^2^	18	20/1000	St.TG; Au – PMMA/Al	68
**BBL**	4.00	—	0.03–0.10	—	—	25/500	Co.BG; SiO_2_/HMDS/Au	51
**P(BTimR)**	3.47	12.7/27.1	3.71	10^6^	25	50/5000	St.TG; Au/CsF – CYTOP/Au	124
**PCNI-BTI**	3.78	26.2/36.7	0.13	10^4^	35	—/5000	St.TG; Au – CYTOP/Al	125
**PDTzTI**	3.77	7.3/7.7	1.61	>10^7^	24	50/—	St.TG; Au – CYTOP/Al	89

a Where values aren’t reported directly in the text, these are inferred from given transfer plots.

b EA is an estimation of the LUMO, although neglects the electron binding energy.

**Table 7:** A selection of other n-type OTFT materials with high reported performance (selection criteria of electron mobility greater than 0.1 cm^2^ V^−1^ s^−1^ and only including ambipolar materials where the n-type performance exceeds p-type), summarising their electron affinity (EA), weight and number average molecular weight (*M*_W_/*M*_n_), maximum reported electron mobility (*μ*_e_), ratio of on to off current (*I*_ON_/*I*_OFF_)^a^, threshold voltage (*V*_T_)^a^, OTFT channel length and width (*L*/*W*) used and summary of device structure. Where: coplanar (co.), staggered (st.), bottom gate (BG), top gate (TG); silicon oxide (SiO_2_), poly(methylmethacrylate) (PMMA), hexamethyldisilazane (HMDS), octyltrichlorosilane (OTS8); gold (Au), aluminium (Al); “—” represents the polymer layer

Polymer	EA^b^ (eV)	*M*_n_/*M*_w_ (kDa)	*μ*_e_ (cm^2^ V^−1^ s^−1^)	*I*_ON_/*I*_OFF_	*V*_T_ (V)	Channel *L*/*W* (μm)	Device structure	Ref.
**PPPyr-Cl**	4.00	15.6/23.4	3.40	20	8.4	10/10 000	Co.BG; SiO_2_/Au/HMDS	130
**PBFI-T**	3.80	47.5/174.8	0.30	>10^5^	25	100/1000	St.BG; SiO_2_/OTS8 – Ag	10
**PDIC8-EB**	3.90	66.9/282.3	0.10	20	8	40/800	Co.BG; SiO_2_/Au/HMDS	131
**PIDOBT-TT**	4.13	37.8/88.8	0.29	10^5^	9	80/5600	St.TG; Au/Ba(OH)_2_ – PMMA/Al	132
**PIDOTT-TT**	4.03	28.6/64.6	0.38	10^5^	8	80/5600	St.TG; Au/Ba(OH)_2_ – PMMA/Al	132
**PIDOTT-BT**	4.05	19.8/46.1	0.45	10^5^	5	80/5600	St.TG; Au/Ba(OH)_2_ – PMMA/Al	132

a Where values aren’t reported directly in the text, these are inferred from given transfer plots.

b EA is an estimation of the LUMO, although neglects the electron binding energy.

In n-type OTFTs, misalignment of the workfunction of the source and drain electrodes with the LUMO level of the OSC results in a barrier to electron injection into/extraction from the n-type OSC.^90^ As the LUMO of OSCs varies, it is therefore difficult to directly compare if the transport properties of the OSC itself have been improved by structural modifications or simply the charge extraction from the material in the device. To facilitate electron injection, such barriers must be minimised, which can be done by careful selection of the source/drain contact material or the addition of self-assembled monolayers (SAMs) at the interface.^91,92^ Surface modifications are also employed to reduce charge trapping and enhance the order of the n-type OSCs when deposited. By improving the interfaces within the OTFT, mobility is maximised.^93^ The minimum gate voltage required to fill trap states and produce mobile charge carriers is the threshold voltage (*V*_T_).

Fig. 5(b) illustrates the operation of an n-type OTFT, where a positive gate bias (*V*_g_) is applied, polarising the dielectric layer and resulting in the formation of a thin interfacial electron accumulation layer in the OSC, through which a drain current (*I*_D_) flows when a voltage is applied between the source and drain electrodes (*V*_d_). The OTFT *I*_d_ as a function of *V*_g_ at constant *V*_d_, is referred to as the transfer characteristics, and from these, mobility can be determined in either the linear (*V*_d_ < *V*_g_ − *V*_T_) or saturation (*V*_d_ ≥ *V*_g_ − *V*_T_) regime (eqn (1) and (2)), where the mobility is dependent on channel length (*L*), width (*W*) and dielectric capacitance (*C*).^83^ Importantly, this mobility is not the intrinsic mobility of the OSC on which the OTFT is based and typically mobilities determined in the saturation regime are higher.1
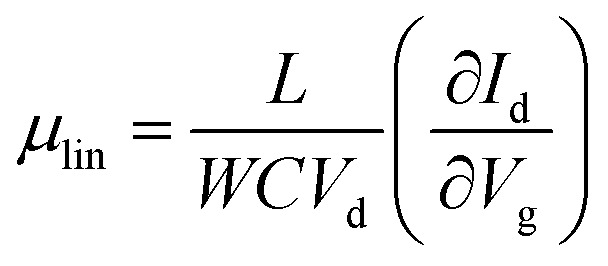
2
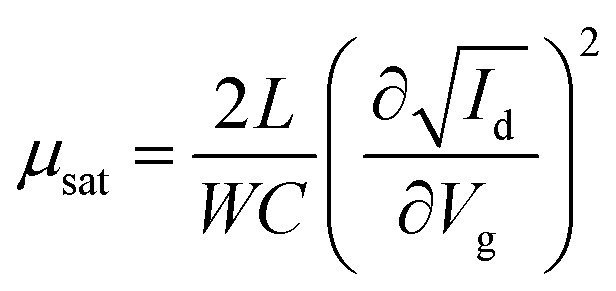
Some of the highest electron mobilities reported use device configurations with channel lengths of 10 μm or less. These short channels have been associated with mobility overestimations, as for short channels, the impact of contact resistance on the current through the channel is proportionally more. Of all the n-type applications for OSCs discussed here, problems with extracting meaningful mobility values from the devices are best understood for OTFTs. It is widely accepted that improper analysis of OTFT transfer characteristics can lead to overestimations of mobility and several works have discussed this in depth.^41,83^ The overestimation of mobility values has distorted the OTFT field making comparison of materials difficult and also hindering the development of promising new materials.

Tables 1–7 present the performance of OSCs in n-type OTFTs for which an electron mobility >0.1 cm^2^ V^−1^ s^−1^ has been reported, specifically where the electron mobility significantly dominates over any hole mobility also reported. In these tables, the OTFT architecture and channel lengths used are summarised to put in context the values of mobility extracted from the OTFTs. The values stated in this review are the highest reported for each material in literature, irrespective of testing conditions or device architecture and geometry. The values have not been normalised and non-ideal output characteristics have not been accounted for or discussed.

### 2.1. Naphthalenediimide (NDI) derivatives

NDI units are present in many of the highest performing polymeric OSCs for n-type OTFTs. The planar bicyclic conjugated system containing two electron-withdrawing imide groups leads to strong π–π stacking interactions, beneficial for charge transport. In addition, functionalisation of the imide units with alkyl chains for example, allows control over the processability and crystallisation of NDI based polymers. Compared with other n-type materials NDI containing polymers have demonstrated good stability in OTFTs operated under ambient conditions, for example OTFTs based on **P(NDI2OD-T2)**, more commonly known as the commercially available **N2200** and one of the most studied materials for n-type OTFT applications, maintain their mobility over several weeks and under a relative humidity of over 50%.^94^ Across a variety of top-gate OTFT architectures with different dielectrics, **P(NDI2OD-T2)** consistently delivers mobilities of between 0.1–0.85 cm^2^ V^−1^ s^−1^.^94^ On top of this, highly oriented films of **P(NDI2OD-T2)** deposited by bar-coating pre-aggregated solutions, where the fibrillar network of OSC was aligned parallel with the OTFT channel, have led to an impressive maximum electron mobility of 6.4 cm^2^ V^−1^ s^−1^.^95^

**Fig. 6:**
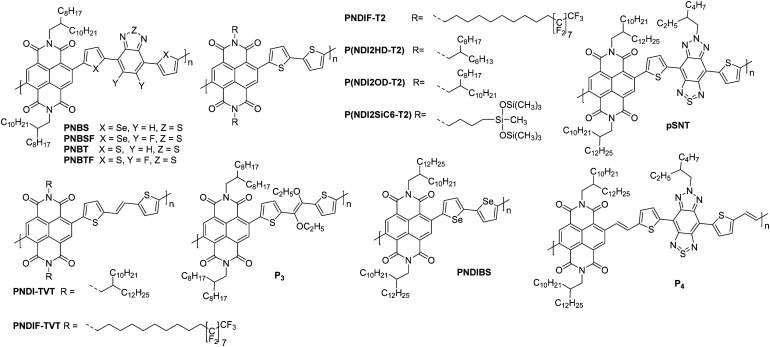
Chemical structures of reported OTFT polymeric materials containing NDI derivatives, including their published synonyms.

The comparison of **P(NDI2HD-T2)** and **P(NDI2OD-T2)** (Fig. 6) considers the impact of sidechain engineering on the performance of the polymer, whilst retaining the same NDI-T2 polymer backbone. The performance of **N2200** was improved upon by changing the sidechains for a shorter branched alkyl alternative, reducing the number of carbons in the chain by four, to give **P(NDI2HD-T2)**, which resulted in a 50% increase in the highest reported mobility for **N2200** at the time (1.22 cm^2^ V^−1^ s^−1^), to achieve a mobility of 1.90 cm^2^ V^−1^ s^−1^ (Table 1).^96^ This simple structural change caused the microstructure to become significantly more crystalline, with a much higher melting enthalpy than **N2200**, comparable to that of highly crystalline **P3HT**.^97,98^ This trend continues when comparing the π–π stacking distance, which shortens as the alkyl chain length is decreased. These combined factors result in a significant increase in the mobility recorded for **P(NDI2HD-T2)** through a simple sidechain modification.

The second NDI series for comparison is **PNBSF**, **PNBS**, **PNBTF** and **PNBT** (Fig. 6), where **PNBSF** is the highest performing n-type material of the series, with good stability in air (Table 1).^62^ Two polymers, **PNBT** and **PNBTF** contain the more traditional thiophene heterocycles flanking the benzothiazole (BT) unit, while the remaining three comprise selenophene moieties. The introduction of a selenium atom brings with it the improved orbital overlap induced by the larger p-orbital, which can facilitate improved electron transport, as such both selenium-containing polymers have higher electron mobilities than those containing thiophenes (Fig. 4).^99,100^ This is due to the enlargement of the heteroatom orbitals moving down the group, which becomes decreasingly well matched with the size of the neighbouring carbon orbitals, worsening orbital overlap, increasing the quinoidal character of the molecule and the energy of the double bond. Furthermore, the selenophene unit introduces excellent film-forming abilities, which can be rationalised through the enhanced interchain heteroatom–heteroatom interactions, possible due to the larger size of selenium orbitals.^62,99^ Another clear extension to this design strategy is the transition from a standard BT unit, to a difluorinated BT unit, which causes a transition from ambipolar transport properties of **PNBS** and **PNBT** to the unipolar electron transport exhibited by **PNBSF** and **PNBTF** (Fig. 6). Fluorination results in a deepening of the LUMO, which can lead to improved operational stability in air.^62^ The introduction of selenium and fluorine atoms does however add synthetic and processing challenges, where the particularly strong intermolecular interactions create solubility issues, limiting them to processing in hot chlorobenzene, while **PNBT** is readily soluble at room temperature in most chlorinated solvents.^62^

Whilst **P(NDI2SiC6-T2)** does not exhibit a particularly notable electron mobility (1.04 cm^2^ V^−1^ s^−1^; Table 1), the chemical design provides an interesting discussion point, as it demonstrates a hybrid material which exhibits advantages of both organic and inorganic properties.^101^ The selected sidechain constitutes an alkyl spacer, capped with a siloxane group, which enables the polymer morphology to be controlled through variation of the solvent, likely due to solubility induced pre-aggregation. For example, casting a film of **P(NDI2SiC6-T2)** from chloroform results in a mixed edge-on and face-on orientation, whilst casting from 1-chloronaphthalene produces a film that is almost exclusively edge-on, with a much more amorphous microstructure.^101^**P(NDI2SiC6-T2)** only displays an out-of-plane (010) π–π stacking reflection in the grazing incidence X-ray diffraction (GIXD) pattern when cast from chloroform, so these interactions cannot be quantified for the exclusively edge-on microstructure. This is supported by the improved electron mobility for the chloroform cast films (1.04 cm^2^ V^−1^s^−1^) when compared to the 1-chloronaphthalene cast films (which has a maximum mobility of 0.85 cm^2^ V^−1^ s^−1^).^101^ Having this control of the microstructure of a polymer, and therefore the charge carrier mobility, simply through changing the processing solvent and conditions, is an attractive proposition.

The concept of modifying the sidechain to alter the microstructure of the material is further demonstrated with **PNDIF-T2**, where a semifluoroalkyl sidechain is utilised.^53^ These sidechains offer strong self-organisation on account of the fluorophobic interactions commonly found in heavily fluorinated molecules, which creates large areas of crystallinity and long-range order, as evidenced by the GIXD pattern which shows a well-defined (*h*00) peak up to the fifth order for the out-of-plane direction and a prominent (010) reflection for the in-plane direction. This coexisting face-on and edge-on arrangement is created by the rigid polymer backbones and is reflected in the impressive electron mobility of 3.93 cm^2^ V^−1^ s^−1^, with good stability in air (Table 1).^53^

Polymers **PNDIF–TVT**, **PNDI–TVT** and **P3** all employ a thienylene–vinylene–thienylene (TVT) electron rich comonomer unit, which is thought to extend the conjugation length of the monomer unit and contribute to improved crystallinity of the backbone (Fig. 6).^52,53,102^ Whilst these polymers are not a comparable series due to the multiple variables, it can be noted that the TVT comonomer appears to deepen the LUMO compared to the T2 moiety, likely due to the extension of the conjugated unit, with these polymers all offering EAs of 4 eV (Table 1). The final two NDI derivatives for discussion are **pSNT** and **P4**, the latter of which exploits the vinylene group to enhance backbone planarity (shown by DFT calculations) through interactions of the vinyl protons with the carbonyl oxygen on the NDI unit.^105^ This, combined with the benzobisthiadiazole (SN), results in a remarkable electron mobility of 7.16 cm^2^ V^−1^ s^−1^.^105^

The NDI derivatives are the largest class of electron transporting OTFT materials and offer some clever design solutions to ensure unipolar transport and overcome amorphous microstructures, with many NDI derivatives displaying stability in air. The main design strategies considered here are those of heteroatom substitution^62,84^ with the aim of improving LUMO delocalisation across the entire polymer backbone rather than its typical localised state on the electron deficient monomer, and sidechain engineering to shorten the sidechain^96^ or introduce halogen atoms to improve the crystallinity of the microstructure through better π–π stacking and self-organisation of the material.^53^

### 2.2. Naphthodithiophenediimide (NDTI) derivatives

Naphthodithiophenediimide (NDTI) polymer derivatives, contain the NDI unit and introduce fused thiophenes to the core to extend the conjugation length, in the hope of improving the electron transport properties. The NDTI unit was first reported in 2013,^106^ and has since been explored with a variety of comonomers and sidechains.^58,107,108^ The closest comparison that can be drawn between the NDI and the NDTI unit is with the polymers **P(NDI2OD-T2)** and **PNDTI-BT-DT**, which aside from the additional fused thiophenes, only differs by two carbon atoms on each sidechain. The NDTI unit has the desired effect of increasing the EA from 3.91 eV to 4.40 eV (Fig. 4).^94,106^ Whilst this has not yet led to an improved electron mobility, DFT calculations indicate a significantly more planar backbone for NDTI derivatives compared to their NDI counterparts.^59,106,109^

**Fig. 7:**
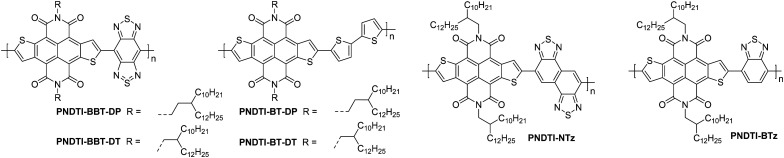
Chemical structures of reported OTFT polymeric materials containing NDTI derivatives, including their published synonyms.

The first chemical design point to note here is the structure of **PNDTI-BT-DP** compared to **PNDTI-BT-DT**,^110^ and **PNDTI-BTT-DP**, compared to **PNDTI-BTT-DT** (Fig. 7), where each of the pair has the same structure aside from an extra carbon before the branch point of the sidechains. In both cases, this has the effect of pushing the alkyl chains further from the polymer backbone, and increases the electron mobility, for example **PNDTI-BTT-DP** shows a three-fold increase from 0.096 cm^2^ V^−1^ s^−1^ of **PNDTI-BTT-DT** to 0.31 cm^2^ V^−1^ s^−1^ (Table 2).^58^ This can be explained by examining the polymers with X-ray diffraction (XRD), which shows an in-plane π–π stacking peak for **PNDTI-BTT-DP**, whereas this is not present for **PNDTI-BTT-DT**. Notably, **PNDTI-BTT-DT** also displays a broad peak assignable to out-of-plane π–π stacking, indicating there may be some face-on orientation in this film, which disrupts the crystalline order of the microstructure and worsens the electron mobility.^58^

The other comparison with these relatively high performing NDTI derivatives is between **PDNTI-BTz** and **PDNTI-NTz** (Fig. 7).^59^ These polymers present the same NDTI unit and sidechains, with the distinguishing feature being the comonomer as either benzo[*c*][1,2,5]thiadiazole (BTz) or naphtho[1,2-*c*:5,6-*c*′]bis[1,2,5]thiadiazole (NTz). In the reporting of this polymer series, other comonomers were used, however they either produced low performance (vinylene), or ambipolar polymers (thienylenevinylene and naphtho[1,2-*b*:5,6-*b*′]-dithiophene).^59^ It was initially thought that the use of a smaller comonomer, such as vinylene, would be advantageous in promoting the orbital overlap with the LUMO, allowing it to delocalise more than usual. However, this picture was not validated, as the largest comonomer actually exhibits the highest mobility (Table 2). This, as before, can be explained by examining the GIXD pattern, where **PDNTI-NTz** has the most crystalline structure, clearly displaying both an edge-on orientation and in-plane π-stacking.^59^

NDTI derivatives have not yet outperformed the heavily studied NDI derivatives. The underlying design principle with these polymers is to increase the backbone rigidity and create polymers with more long-range order and larger regions of crystallinity, but this remains to be demonstrated.

### 2.3. Polylactam/lactone derivatives

**Fig. 8:**
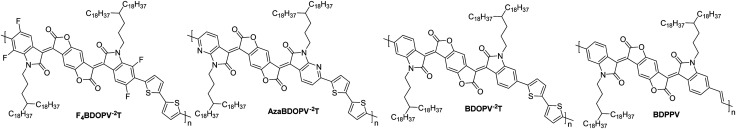
Chemical structures of reported OTFT polymeric materials containing polylactam/lactone derivatives, including their published synonyms.

Polylactam/lactone polymers have been demonstrated to exhibit promising electron mobilities. One example is **BDPPV** (Fig. 8), which utilises benzodifurandione (BD), an electron deficient unit, which aims to lower the LUMO whilst also taking advantage of the carbonyl groups, which can form intramolecular non-covalent short contacts with phenyl protons to “lock” the double bonds into the *trans* conformation (Fig. 4).^87^ Combining this with 4-octadecyldocosyl sidechains,^87^ which allows for excellent interchain π–π stacking as the branch point is further from the polymer backbone,^111^ a design strategy which has been shown to improve mobility (Table 3). The electron mobility of **BDPPV** was further improved with **BDOPV-2T** (Fig. 8), which introduces the donor comonomer, bithiophene. Interestingly, when devices were fabricated in a glovebox and tested in ambient conditions, the hole mobility was minimal, however when fabricated in ambient conditions, hole mobility increased to a maximum of 0.47 cm^2^ V^−1^ s^−1^, giving the material ambipolar properties.^86^

The next polymer in the series, **AzaBDOPV** (Fig. 8), builds on this D–A polylactam/lactone derivative and incorporates an additional nitrogen into the polylactam/lactone core, transforming the phenyl into a pyridine derivative.^60^ These sp^2^ nitrogen atoms create a more electron deficient acceptor unit, which increases the EA to 4.37 eV (Fig. 4). This facilitates electron injection, increases the mobility by two-fold to 3.22 cm^2^ V^−1^ s^−1^ in ambient conditions and unsurprisingly, it also removes the observation of ambipolar transport for this material (Table 3).^60^ The final polymer, **F4BDOPV-2T** (Fig. 8), also aims to improve on **BDOPV-2T** with the introduction of two fluorine atoms on the phenyl rings.^85^ These have the dual purpose of both reducing the electron density of the polylactam/lactone core and increasing the planarity of the backbone *via* further intramolecular non-covalent short contacts between one fluorine atom and a proton on the bithiophene unit, and the strong non-covalent F–S interaction. These interactions can be visualised by examining the phenyl–thienyl dihedral angle, which decreases from 21.9° in **BDOPV-2T**, to 9.6° in **F4BDOPV-2T**.^85^ This must be on account of the interactions because the fluorine atom itself has an atomic radius that is almost 25% larger than hydrogen.^112^ These combined effects increase the EA to 4.32 eV (Table 3).

The strategic design surrounding these polylactam/lactone derivatives include introducing an electron-donating comonomer (typically bithiophene), utilising electron-withdrawing functionalities (such as fluorine atoms appended to^85^ or nitrogen atoms embedded into the backbone)^60^ to deepen the LUMO of the polymer and improve electron injection, and focussing on how to create backbone planarity through intramolecular interactions, including non-covalent short contacts (Fig. 4). Due to the electron deficient backbones, and in turn the low-lying LUMOs of polylactam/lactone derivatives in this series, they all produce air-stable n-type devices, with some of the highest mobilities reported for electron transporting OTFTs.^85^

### 2.4. Isoindigo (IIG) derivatives

The isoindigo (IIG) unit makes use of an exocyclic double bond between the two five membered rings which acts to planarise the backbone. The dipole of the carbonyl groups are able to promote intermolecular interactions and also act to deepen the LUMO level, increasing n-type stability and improving electron injection.^113,114^

**Fig. 9:**
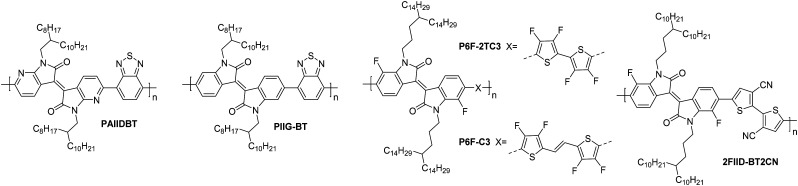
Chemical structures of reported OTFT polymeric materials containing isoindigo (IIG) derivatives, including their published synonyms.

The lowest performing of the isoindigo polymers reported in Table 7, **PIIG-BT**, was reported alongside **PIIG-TPD**, which displays the same IIG core and sidechains, and a thienopyrroledione comonomer unit (Fig. 9).^65^ The mobility improvement for **PIIG-BT** compared to **PIIG-TPD** is explored by examining the polymers with atomic force microscopy (AFM), where both films formed densely interconnected nanofibrillar domains, with the grains in **PIIG-BT** over double the size of those in **PIIG-TPD**.^65^ It is believed that this gives **PIIG-BT** higher electron mobilities, owing to the ability of the material to form much larger crystalline domains.^65^

**PIIG-BT** is then further improved by mimicking the approach taken with the polylactam/lactone polymers, through the introduction of nitrogen atoms into the phenyl rings, to form **PAIIDBT**, which results in the IIG unit becoming more electron deficient and increasing the EA from 3.54^65^ to 4.10 eV,^64^ leading to a five times increase in mobility (Table 4). The difference with this design approach however, is that these materials are an acceptor–acceptor (A–A) copolymer, constituting two electron deficient monomers. This approach aims to lower the LUMO to improve ambient stability and potentially allow for more dense packing of polymer chains (Fig. 4).^117^ Use of A–A motifs also results in a deepening of the HOMO, increasing the energetic barrier to hole injection, bringing the potential benefit of lowering OTFT off-currents.^89^ The use of traditional D–A copolymers is a well-established synthetic plan to improve performance, however the relatively new field of A–A copolymers is still worth studying for the prospect of developing high performing n-type OTFT materials.

### 2.5. Diketopyrrolopyrrole (DPP) derivatives

The diketopyrrolopyrrole (DPP) unit is electron deficient as a result of the carbonyl groups on the conjugated lactams, and has been commonly employed in D–A polymers for p-type transport.^118^ However, it has also been shown, when combined with another acceptor unit, to produce polymers that are able to support electron transport.

**Fig. 10:**
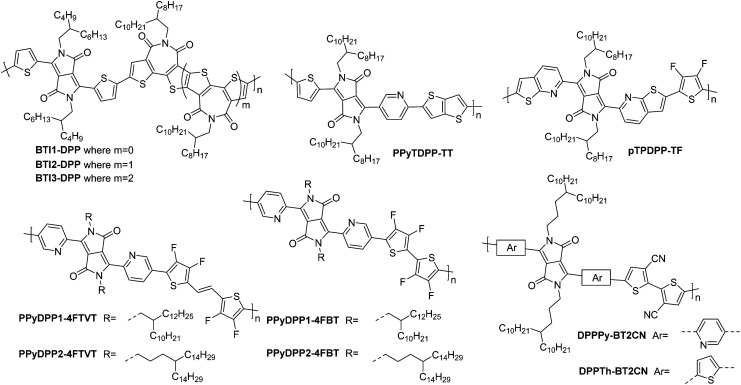
Chemical structures of reported OTFT polymeric materials containing diketopyrrolopyrrole (DPP) derivatives, including their published synonyms.

The first series compares **BTI1-DPP**, **BTI2-DPP** and **BTI3-DPP** (Fig. 10), whereby the BTI unit extends in length by fusing another BTI unit through the terminal thiophene.^119^ With each additional BTI unit, the electron acceptor character increases, and the EA increases to 3.46 eV for **BTI3-DPP**, from 3.34 eV for **BTI1-DPP**, however backbone planarity is compromised, with the dihedral angle increasing from ∼3° to 10°, as predicted by DFT calculations.^119^ The highest electron mobility is observed for **BTI2-DPP** (0.48 cm^2^ V^−1^ s^−1^; Table 5), where there is a careful balance between deepening the LUMO sufficiently to support electron injection and ensuring planarity of the backbone is maintained.^119^

To assess the influence of the comonomer, the sidechain length and the branching position on n-type performance, a series of four DPP polymers incorporating fluorine atoms were investigated, namely **PPyDPP1-4FBT**, **PPyDPP2-4FBT**, **PPyDPP1-4FTVT** and **PPyDPP2-4FTVT** (Fig. 10).^121^ All polymers exhibit electron mobilities above 1.0 cm^2^ V^−1^ s^−1^, with the highest performing, **PPyDPP2-4FBT**, presenting *μ*_e_ of 2.45 cm^2^ V^−1^ s^−1^ (Table 5).^121^ All polymers show stronger (100) and (200) diffraction peaks by GIWAXs than the non-fluorinated derivative, indicating a higher crystallinity in these materials, which accounts for the impressive electron mobilities. Furthermore, fluorination decreases the π–π stacking distances, from 3.59 Å for **PPyDPP1-BT**, to 3.56 Å for **PPyDPP1-4FBT**.^121^

The final DPP comparison is that of **DPPTh-BT2CN** and **DPPPy-BT2CN**, whereby the flanking units around the DPP are altered from thiophene to pyridine motifs, to investigate the impact on the LUMO level and the electron mobility (Fig. 10).^116^ These, combined with electron-withdrawing cyano functionalities on the T2 comonomer, ensure the LUMOs are deep enough to support n-type behaviour, resulting in electron mobilities of 0.35 and 0.30 cm^2^ V^−1^ s^−1^ respectively (Table 5).^116^ Whilst **DPPTh-BT2CN** displays a low hole mobility, the pyridine derivative suppresses hole injection completely through increasing the ionisation potential (IP, which is an approximation of the HOMO energy level) from 5.41 to 5.83 eV.^116^ This wide selection of DPP polymers clearly shows that this moiety has application in n-type OTFT materials, despite not offering particularly high EAs.

### 2.6. Acceptor–acceptor (A–A) derivatives

**Fig. 11:**
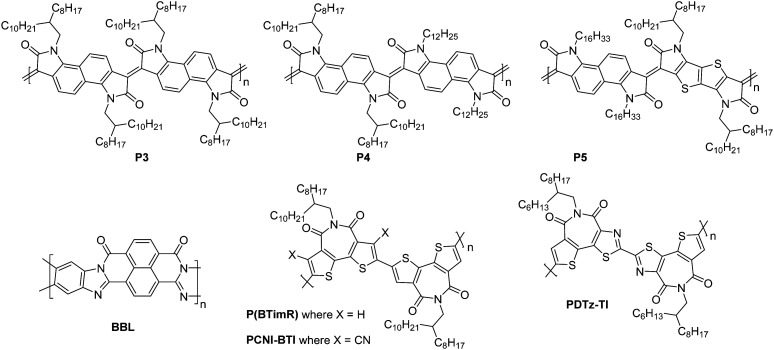
Chemical structures of reported OTFT polymeric materials containing an acceptor–acceptor (A–A) motif, including their published synonyms.

The fully fused rigid polymers take note of the above design strategies, particularly the polylactam/lactone derivatives, to further increase backbone rigidity and the A–A motif of the isoindigo derivatives, whilst also incorporating non-toxic polymerisation conditions and exclusion of precious heavy metals in the synthetic procedures *via* aldol condensation.^36^ The use of bisisatin and bisoxindole monomers in a conformationally locked arrangement, having removed all single carbon–carbon bonds, results in a dihedral angle of 10 to 20° between all aromatic units.^36^ Short intermolecular contacts can be registered by alternating bulky sidechains with shorter linear sidechains,^122^ an approach that was exploited when moving from **P3** to **P4** (Fig. 11).^36^ Optimisation of the processing of **P4**, specifically aligning the polymer backbones parallel to the transistor channel through solution shearing, led to a mobility of 0.2 cm^2^ V^−1^ s^−1^ compared with the initial reported mobility of 0.03 cm^2^ V^−1^ s^−1^ (Table 4).^36,62^ The series was further expanded to **P5**, which alters the aromatic core of one comonomer to a thieno[3,2-*b*]thiophene unit. This has the desired effect of increasing the planarity, with DFT calculations predicting the angle to be 0°. Due to solubility issues, **P5** was synthesised to a low *M*_n_ of 8.3 kDa, which is probably the reason for the lower than expected charge carrier mobility.^123^

The fused polymer, **BBL**, has recently been a pioneering material in the fields of n-type OECTs and OTEs. In OTFTs, an electron mobility of 0.1 cm^2^ V^−1^ s^−1^ has been achieved, which has been attributed to the close π-stacking of the fused backbone (3.51 Å).^51,126^ Thin films of **BBL** have been shown to contain crystalline domains on the order of 50–125 nm and this crystallinity has been suggested to act as a kinetic barrier to prevent oxygen diffusion into the films, thereby enhancing the stability of OTFTs based on **BBL**.^127^

Another A–A example are polymers containing the thiazole imide moiety. Whilst the fused thiophenes of the thiazole imide motif, shown in Fig. 11 as **P(BTimR)**, **PCNI-BTI** and **PDTzTI**, are electron-rich units, the strongly electron-withdrawing imide group overrides the monomer to give it an electron accepting character. The simplest of the series is **P(BTimR)**, which contains only a single repeat unit and exhibits a high electron mobility of 3.71 cm^2^ V^−1^ s^−1^.^124^ This material, however, exhibits a low EA of 3.47 eV, severely limiting the ambient stability. **PDTzTI** has been synthesised to include thiazole motifs, increasing both the EA and IP. However, the EA remains relatively low at 3.77 eV, which still limits the ambient stability of these devices.^89,128^ Cyanation is an effective way to deepen the LUMO level of OSCs, and in this case a series of cyanated **P(BTimR)** derivatives were demonstrated to effectively increase the EA from 3.47 to 3.78 eV, though this was still not fully effective to sufficiently improve ambient stability and did not increase *μ*_e,max_ (Table 6).^125^

### 2.7. Other OTFT n-type polymers

**Fig. 12:**
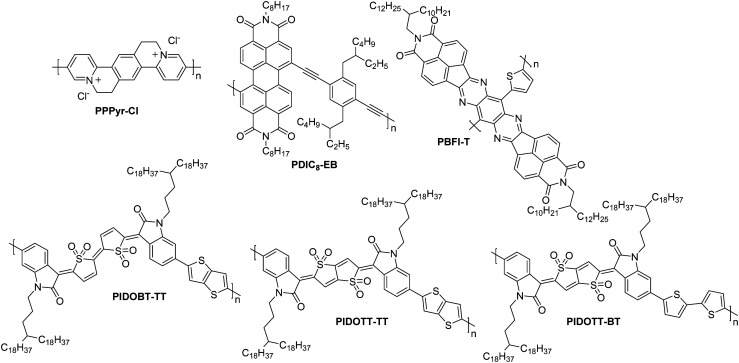
Chemical structures of a selection of the remaining unclassified n-type OTFT polymeric materials, including their published synonyms.

This section presents the key performance metrics of OTFT polymers that did not fall into previous categories, namely those found in **PPPyr-Cl**, **PDIC8-EB** and the PIDO series (Fig. 12).

**PPPyr-Cl** (Fig. 12) demonstrates a cationic backbone structure which affords a high EA of 4.00 eV and an impressive *μ*_e,max_ of 3.40 cm^2^ V^−1^ s^−1^ (Table 7). High gate voltages are required to obtain these mobilities (15–20 V) whereas mobilities at a gate voltage of 5–15 V are 0.24 cm^2^ V^−1^ s^−1^.^129,130^ The approach of utilising a water-soluble ionic structure remains interesting for application in transistors, and could potentially also see uses in OECT devices.

Another polymer which provides a novel design strategy is **PDIC8-EB**, due to its employment of alkyne linkers between the two comonomers (Fig. 12). Without these, the phenyl–phenyl linker has a low quinoidal character and would result in a high degree of twisting along the backbone, reducing crystallinity and electron transport properties. The introduction of the linear alkyne group supports the predisposition of perylene diimide (PDI) units to π-stack, which results in a reasonable OTFT *μ*_e_ of 0.1 cm^2^ V^−1^ s^−1^ (Table 5).^131^

The final series for discussion utilises a novel thienoquinoidal unit flanked with isatin groups. This unit is either in a bithiophene or thienothiophene type arrangement, and coupled with T2 or TT in a D–A motif to present the polymers **PIDOBT-TT**, **PIDOTT-TT** and **PIDOTT-BT** (Fig. 12).^132^ This design strategy employs a sulfone group, which forces a single isomeric structure due to the steric hinderance of the many oxygens in close proximity. These polymers have high EAs above 4.0 eV, resulting in good air stability and reasonable electron mobilities, with the highest performer, **PIDOTT-BT**, recording 0.45 cm^2^ V^−1^ s^−1^ (Table 7).^132^ This can be explained by the planarity of the backbone of this polymer, which has continuously been found to relate to the order of the thin films. Due to the fused nature of the TT unit in the centre of the acceptor monomer of **PIDOTT-BT**, this monomer has a dihedral angle of 0° by X-ray diffraction, enabling short π–π stacking distances of 3.45 Å.^132^ This novel series proves that whilst there is significant focus in the field on optimising pre-existing polymeric materials, there are still alternatives yet to be discovered that offer reasonable OTFT performance.

### 2.8. Summary of OTFT material design considerations

A wide range of design strategies have been discussed for application to n-type OTFT materials. These include heteroatom substitution into the polymer backbone, as seen in the NDI^62^ and IIG^64,65^ derivatives. When the heteroatom has a larger electronegativity than carbon, this has the effect of reducing the electron density in the conjugated pi electron system, thus increasing the electron affinity of the polymer. In the case of using larger chalcogen atoms to replace sulfur for example, the larger p-orbitals provide better overlap thus improving electron transport in this way. The polymer backbone can also be made more electron deficient and thereby more efficient at transporting negative charge by introducing electron-withdrawing groups, such as fluorine atoms (Fig. 4).^85^ To exploit this, sidechain modifications can be employed to alter both the solubility and packing of polymeric OSCs. Varying the length of alkyl chains and utilising either branched or linear alkyls will strongly impact its solubility, molecular weight and packing (with shorter chains resulting in a more crystalline microstructure^96^), however these properties are most greatly affected by the addition of semifluoroalkyl sidechains,^53^ which are able to self-organise into a regime of long-range crystalline order, making electron transport among the highest reported in n-type OTFT devices. The PPV,^60,85–87^ IIG^64,65^ and fused acceptor–acceptor^36^ polymers all exercise strategies to maximise planarity of the backbone, including minimising single bonds, whereby rotation is freely accessible, and intrachain H-bonding between carbonyls and neighbouring hydrogens on the core. These polymers aim to minimise trapping sites through increasing the microstructure order to improve electron transport. The rigid rod systems take this design idea a step further by utilising only electron deficient monomers, in an acceptor–acceptor copolymer.^36^ Whilst this doesn’t provide exceptional mobilities in OTFTs, it is an area for further consideration to increase the EA and improve electron injection.

## 3. n-Type organic electrochemical transistors

### 3.1. Assessing charge transport in n-type organic electrochemical transistors

OECT devices have the ability to transduce a biological signal into a readable electrical signal,^133^ so can be used to monitor biological functions, such as measuring glucose levels in diabetic patients, or monitoring variations in lactate concentrations.^134^ Organic materials can be considered more compatible with biological milieu than inorganics, possessing more similar mechanical properties. For ionic and small molecule sensing, the hydrated, open structure of the semiconducting polymers employed as the active layer allows diffusion of the analyte into the bulk providing unrivalled sensitivity and signal amplification.

**Fig. 13:**
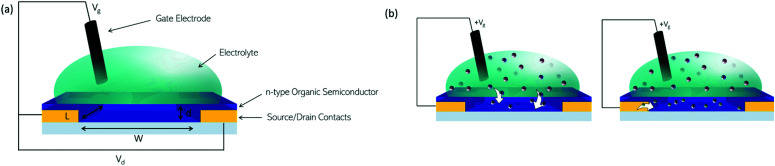
(a) Architecture of an OECT. (b) Illustrations of (left) migration of positive ions from aqueous electrolyte into an n-type organic semiconductor on application of a positive gate bias. (right) Compensation of positive charges in the organic semiconductor film by electrons.

The n-type OSCs applied to OECTs are mixed conductors which allow for electron transport along their conjugated backbones and ionic transport through the bulk of the material (Fig. 13(a)). In an n-type OECT operating in accumulation mode, the current between the source and drain electrodes is modulated by changes in the effective gate bias, which causes an injection of electrons into the volume of the channel, followed by a migration of ions into the bulk OSC layer (Fig. 13(b)). This migration of ions is controlled by either an immersed electrode in the OECT electrolyte or biomolecular activity changing ion gradients in the electrolyte.^135^

The mixed conduction of both electrons and ions by the n-type OSC means that charge transport cannot be modelled in the same way as in OTFTs. One of the first theories developed to model the operation of OECTs was Bernards’ model, which simultaneously applies an electronic and ionic circuit to treat OECTs operating in depletion mode, as opposed to accumulation mode.^135^ Non-uniform charge carrier densities across the channel, contact resistance, swelling of the polymer film on operation and polaron binding energies are further considerations which are taken into account by more developed models.^136^

For OECTs, the slope of change in current as a function of gate bias (eqn (3)), referred to as the transconductance, *g*_m_, is commonly used to compare the properties of OECTs.^39,137^3
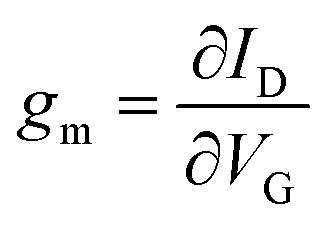
Electron transport in OECTs is through the bulk of the OSC rather than a thin interfacial charge accumulation layer, as is the case in OTFTs. As for OTFTs, the channel length (*L*) and width (*W*) affect the transconductance value extracted from OECTs,^138^ however, the thickness of the OSC layer (*d*) has also been shown to be proportional to the transconductance value extracted.^136^ The channel geometry chosen depends on the desired application of the OECT, but are typically less than 20 μm for screening new materials.^7,139,140^ Transconductance values are often normalised with regards to channel dimensions or drain voltage, however, as they are not an accurate representation of the mixed conductor performance, rather the steady state performance, so far reporting of transconductance values is not standardised across the research field, making a fair comparison of materials difficult.^137^

A metric more relevant to the transport properties of the n-type OSC itself, rather than the OECT device, is the product of mobility (*μ*) and capacitance of the channel per unit volume (C*). This so called [μC*] product considers the device geometry, effective gate voltage (*V*_g_) and threshold voltage (*V*_T_) of the OECT. Using eqn (4), [μC*] can be determined from the OECT transconductance.4
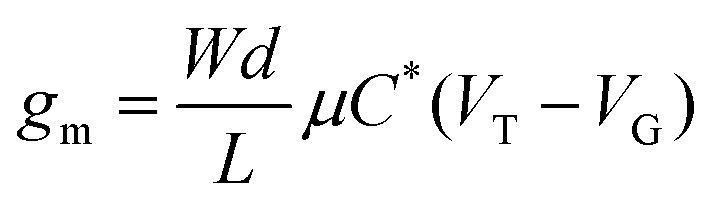
The speed of ion migration is related to the resistance of the electrolyte and C*, thus OECT response time is limited by the speed of migration of ions into the OSC layer. As C* is related to *d*, it follows that OECT response time decreases as channel thickness increases.^39^**p(gNDI-gT2)** acts as a case in point for the importance of a gold-standard figure of merit for OECT materials aside from *g*_m_′, as the *g*_m_′ value reported shows clear device dimension dependence, even when thickness normalised.^81^**p(gNDI-gT2)** recorded a transconductance of 21.7 μS in a device where *W* = 100 and *L* = 10 μm, but a transconductance of 2.72 μS in a 50 × 50 μm device. In both cases, the thickness of the device was 200 nm, so the thickness normalised transconductance terms vary by a factor of 10, simply due to differences in device geometry.^81^

In some cases, the [μC*] product has been decoupled ([*μ*][C*]) by determining mobility through time of flight or impedance spectroscopy measurements. A similar trend in [μC*] derived directly from OECTs and [*μ*][C*] calculated from independent *μ* and C* measurements has been demonstrated, though in general, the calculated [*μ*][C*] product is underestimated relative to the [μC*] product.^137^ Every technique for determining the mobility of disordered OSCs will give slightly different values due to the nature of the systems under investigation and the measurement itself. This discrepancy between some material measurements and the actual application of the device can lead to an overinflation of how useful a material is.

Exposure of OSCs to aqueous electrolyte has been shown to result in the migration of water molecules as well as ions into the film, causing it to swell. The magnitude of this swelling is dependent on the chemical structure and morphology of the OSC and a greater degree of swelling results in a higher volumetric capacitance and thereby increased transconductance.^141,142^ Whilst swelling is beneficial for efficient uptake of ions by the OSC at low operating voltages, excessive OSC swelling has been shown to have a detrimental effect on mobility,^143^ as the crystallinity of the microstructure is disrupted and so the number of pathways for charge transport throughout the polymer are reduced.^133^

### 3.2. Organic electrochemical transistor materials

There has been a recent growth in the development of materials, known as “mixed-conduction” polymers, which offer both electronic and ionic transport, and the majority facilitate this utilising oligo(ethylene glycol) sidechains. These glycol chains provide the dual functionality of solubilising the polymer backbone, and enhancing ion penetration into the bulk of the polymer during electrochemical doping due to their inherent polarity.^142^ This sidechain engineering technique has been reproduced on multiple occasions and remains a proven design strategy to deliver a range of high performing OECT materials.^78,81,142,144,145^

**Fig. 14:**
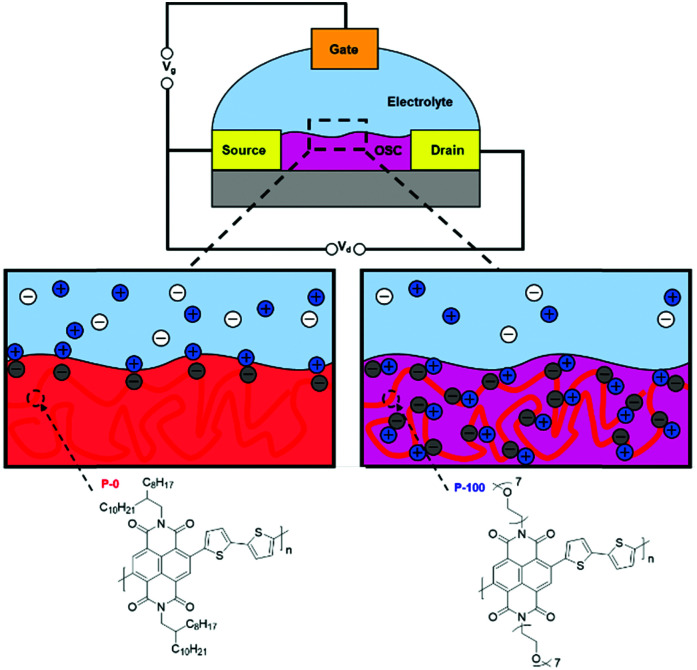
The molecular structures of the all alkyl OTFT material (**P-0**) and all glycol OECT material (**P-100**) and schematic illustrations of their operation. Cations are depicted in blue, anions in white and electrons throughout the polymer backbone are grey. Adapted from literature.^80,142^

Through sidechain engineering, changing the ratio of alkyl and glycol sidechains enables the NDI-T2 polymer backbone to be tailored to two-dimensional OTFT operation or to optimum OECT operation with three-dimensional charge transport (Fig. 14).^142^ This n-type backbone, with all alkyl sidechains, performs well in OTFT devices, but its hydrophobicity inhibits its ability to operate as the mixed transport layer in an OECT. By introducing a controlled ratio of glycol chains, *i.e.* an alkyl/glycol sidechain random copolymer, ionic transport can be facilitated, allowing the polymer to operate as an OECT, when the percentage of glycol chains reaches 75% (Fig. 14).^142^ The optimal ratio for this backbone was found to be 90% glycol and 10% alkyl sidechains, resulting in the polymer, **P-90**.^142^**P-90** is favoured for use with enzymes, such as the work with glucose oxidase (GOx), which requires a suitable surface interaction between the OSC and the enzyme.^146^ It has been observed that the enzyme adsorption on the surface of the thin film is quite sensitive to the 90% glycol ratio, demonstrating that the sidechains not only impact the performance of a material in OECTs, but also affect the devices potential applications based on interactions with the desired biological media.

The particular difficulty with electron transporting OECT materials is their requirement to operate in ambient conditions, meaning their electron polaron must be stable to oxygen and water. As previously stated (Fig. 2), a deep LUMO level is required to aid stability, as well as a low threshold voltage to ensure the device operates at its optimum performance within applied voltage range of 0.89 to −1.23 V, to avoid the oxidation of water or the reduction of oxygen respectively.^81^

Whilst a material can be inherently ambipolar, it has been observed that when sweeping the voltage in one direction to achieve one type of charge transport, then sweeping in the opposite direction, the stability of the material in both directions is limited upon repeating the cycling. Mitigation against this degradation can be achieved by suppressing hole injection with a low-lying HOMO.^4,147,148^

**Fig. 15:**
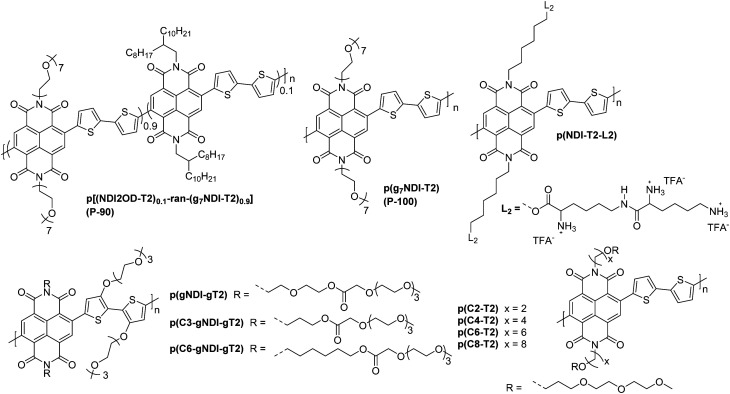
Chemical structures of reported OECT polymeric materials containing NDI derivatives, including their published synonyms.

**Fig. 16:**
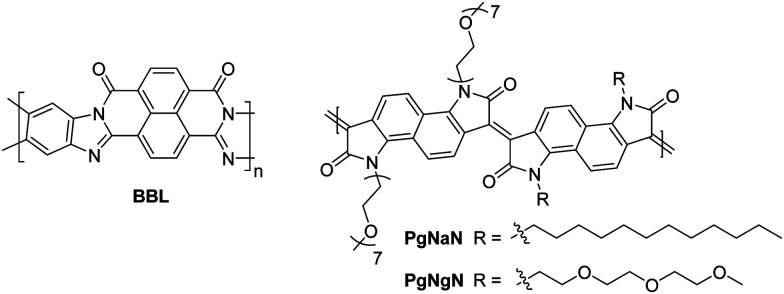
Chemical structures of reported OECT polymeric materials utilising a fully fused acceptor–acceptor derivatives, including their published synonyms.

Taking the figures of merit discussed in Section 3.1 into consideration, it is quite clear that a material can be deemed high performing based on a number of different parameters. These materials fall into two main categories: naphthalenediimide (NDI) derivatives (Fig. 15),^142^ and fully fused A–A polymeric backbones (Fig. 16), in both cases building on conjugated backbones widely utilised in polymers designed for n-type OTFTs.^7,149^ The key properties and common figures of merit for a comprehensive selection of OECT n-type polymers are set out in Table 8.

**Table 8:** The electron affinity (EA), number average and weight average molecular weights (*M*_n_ and *M*_w_), the electron mobility (*μ*_e_), volumetric capacitance (C*), the gold-standard figure of merit, [μC*] and thickness normalised transconductance (*g*_m_′)

Material	EA^a^ (eV)	*M*_n_/*M*_w_ (kDa)	*μ*_e_ (cm^2^ V^−1^ s^−1^)	C* (F cm^−3^)	[μC*] (F cm^−1^ V^−1^ s^−1^)	*g*_m_′ g (S cm^−1^)	Ref.
**P-90**	4.23^b^	7.8/12.4^e^	2.38 × 10^−4^	198	—	0.210	142
**P-100**	4.17^b^	7.2/9.0^e^	1.96 × 10^−4^	192	—	0.204	142
**p(NDI-T2-L2)**	4.50^b^	Not reported	—	95	0.31	0.520	150
**p(gNDI-gT2)**	4.12^c^	16.8/50.1^e^	1.00 × 10^−5^	397	—	1.085	81 and 151
**p(C3-gNDI-gT2)**	4.10^c^	32.4/73.5^e^	—	72	0.13	0.34	151
**p(C6-gNDI-gT2)**	4.00^c^	20.2/54.1^e^	—	59	0.16	0.37	151
**p(C2-T2)**	4.10^b^	18.8^f^	3.97 × 10^−4^	492	0.20	0.40	152
**p(C4-T2)**	4.10^b^	11.3^f^	1.90 × 10^−3^	158	0.30	0.63	152
**p(C6-T2)**	4.20^b^	25.0^f^	4.74 × 10^−3^	272	1.29	2.28	152
**p(C8-T2)**	4.20^b^	14.9^f^	3.76 × 10^−4^	342	0.13	0.15	152
**BBL**	4.00^d^	Not reported	2.14 × 10^−3^	731	1.99	0.815	126
**PgNaN**	4.28^c^	20.7/162.1^e^	6.50 × 10^−3^	100	0.66	0.212	149
**PgNgN**	4.35^c^	8.7/19.3^e^	1.89 × 10^−4^	239	0.04	0.007	149

a EA is an estimation of the LUMO, although neglects the electron binding energy.

b These EA values were measured by subtracting the optical bandgap from the IP (measured by PESA).

c These EA values were measured with cyclic voltammetry in 0.1 M TBAPF_6_ acetonitrile solution, using the onset of reduction to calculate the EA.

d Method of obtaining this range was unspecified.

e These molecular weight values were determined by GPC.

f These molecular weight values were determined by MALDI-TOF.

g *g*_m_′ represents the thickness normalised transconductance; where this was not explicitly reported, it has been calculated by dividing the transconductance by the thickness.

#### 3.2.1. Naphthalenediimide (NDI) derivatives

The NDI-derivative, **P-90** (Fig. 15), is a high performing n-type OECT material with good signal amplification (*g*_m_′ = 0.210 S cm^−1^) and as such has been used in a number of bioelectronic applications.^146,153^ However, the molecular weight of the original batch of **P-90** was low (*M*_n_ of 7.8 kDa), likely leading to statistical variations in individual polymer chain compositions, with subsequent batches giving a *g*_m_′ of 0.06 S cm^−1^.^154^ It does however appear that a small proportion of alkyl sidechains are beneficial to the performance of OECT devices, particularly when examining the electron mobility, *e.g.***P-90**: *μ*_e_ = 2.38 × 10^−4^ cm^2^ V^−1^ s^−1^, while **P-100**: *μ*_e_ = 1.96 × 10^−4^ cm^2^ V^−1^ s^−1^.^142^ The hydrophobic alkyl chains are also thought to act to reduce the swelling of the film during its doped state, thus retaining crystallinity within the microstructure to efficiently transport electrons through the material, as previously demonstrated with p-type OECT OSCs.^143,155^

As in the case of OTFTs, optimisation of polymer processing and introducing n-type dopants have delivered enhanced OECT performance, for example, solvent engineering has been shown to increase transconductance by up to three-fold.^156^ Films of **P-90** are typically spin-cast from pure chloroform (pristine film), however one study utilised chloroform with increasing percentages of acetone, from 5–20 vol%, with peak performance observed at 15% acetone to give a [μC*] product of 0.057 F cm^−1^ V^−1^ s^−1^ (from 0.0188 F cm^−1^ V^−1^ s^−1^).^156^ This is explained by an altering of the morphology due to a change in solubility of the polymer induced when introducing a poorer solvent (acetone), which causes larger regions of the films to be crystalline, due to greater aggregation in the solution. Above 15% acetone, it is thought that the solution became too aggregated, resulting in films with a high degree of crystallinity, which then hinders efficient ion transport due to a lack of amorphous regions.^156^ Another approach to boost the amplification ability of **P-90** based OECTs encompasses the use of the n-dopant, tetra-*n*-butylammonium fluoride (**TBAF**).^154^ Doping had not been utilised in n-type OECT materials, and with an optimal addition of 40 mol% of the dopant, this method has provided not only an extremely impressive *g*_m_′ of 0.910 S cm^−1^ but also good stability, with no changes to peak current or transconductance when pulsed at 0.5 V for 4.5 hours.^154^

Further studies have been carried out to examine the effect of using a hybrid sidechain, composed of both aliphatic and ethylene glycol motifs.^151,152^ The first of these studies takes the high performing material **p(gNDI-gT2)** and introduces an alkyl spacer between the NDI unit and the ethylene glycol chain in order to reduce detrimental swelling properties observed upon doping. This strategy is verified by comparing the C* values of the original **p(gNDI-gT2)** polymer (221 F cm^−1^) with the propyl and hexyl spacer materials (72 and 59 F cm^−1^; **p(C3-gNDI-gT2)** and **p(C6-gNDI-gT2)** respectively; Table 8 and Fig. 15).^151^ As expected, when the hydrophobic alkyl content is increased in these materials, the C* value decreases on account of fewer ions being stored by the polymer, as determined by Electrochemical Quartz Crystal Microbalance with Dissipation (EQCM-D).^151^ This reduced swelling also contributes to a more stable current response, with no decline in the current when switching on and off for 2 hours for **p(C6-gNDI-gT2)** at the gate voltage where peak transconductance is measured (0.6 V), which proves useful for practical application in bioelectronic devices.^151^ The study reports that no accurate values could be obtained for the electron mobilities of these materials due to the low currents observed, however the increase in the [μC*] product observed when lengthening the alkyl spacer from zero (0.06 F cm^−1^ V^−1^ s^−1^) to six (0.16 F cm^−1^ V^−1^ s^−1^) implies that electron mobility must be increasing.^151^

The second spacer study utilises a similar approach, aiming to find the optimum alkyl spacer length for highest OECT performance, varying the spacer length from C2 to C8 (namely **p(C2-T2)**, **p(C4-T2)**, **p(C6-T2)** and **p(C8-T2)**) (Fig. 15).^152^ The OECT performance, measured by both *g*_m_′ and [μC*], increases from **p(C2-T2)** (0.40 S cm^−1^) through to **p(C6-T2)** (2.28 S cm^−1^) then drops for the octyl spacer unit (Table 8). As speculated in the previous study, the electron mobility here is observed to increase with alkyl spacer length, peaking for the hexyl spacer unit, and explaining the trend seen both with the [μC*] product and normalised transconductance, which presents **p(C6-T2)** as the current highest performing n-type OECT material based on both transconductance and [μC*].^152^ This is due to the increase in long-range order through the formation of more interconnected crystalline regions.^152^ These design strategies remain relatively straightforward and prevent the need for a random three component polymer, such as **P-90**, to control levels of swelling and optimise morphology to maximise performance.

#### 3.2.2. Fully fused acceptor–acceptor derivatives

In OECT applications, NDI derivatives are the largest class of n-type active layer materials, though other polymeric structures rival their performance, namely **BBL** (Fig. 16). **BBL** was originally reported in 1969,^157^ and has since been shown to have applications in OPVs,^158^ OTEs,^159^ OTFTs^160,161^ and OECTs,^7^ as well as having the ability to be doped by a range of ions.^162^**BBL** is commonly regarded as one of the highest performing n-type OECT materials to date, with a highest reported volumetric capacitance of 930 F cm^−3^, almost three times that of current state of the art materials (Table 8).^7^ This can be explained by the lack of sidechains which enables the material to store charge in closer proximity to the backbone, *i.e.* storing a larger total amount of charge, when compared to the NDI derivatives.^7^**BBL** also offers an improvement on the electron mobility over other previously reported n-type OECT materials, which has been attributed to the rigid nature of the polymer backbone. Combining the C* and *μ*_e_ values results in an impressive transconductance and creates some key design principles for maximising electron transport, namely increasing the backbone planarity and reducing or eliminating the use of sidechains.

A recent study which compared materials, **BBL** and **P-90**, managed to improve on the previously high *g*_m_′ value of ∼0.6 S cm^−1^ with an increase of one third, to realise a *g*_m_′ of 0.815 S cm^−1^.^126^ It should be noted that the key difference in this study compared to the original work with **BBL** in OECT devices is the processing method, which was originally spray coating, and in this work utilised a spin-coating deposition method.^7,126^ This emphasises the importance of selecting the appropriate processing conditions to maximise the performance of a material. This work also produced a [μC*] value of 1.99 F cm^−1^ V^−1^ s^−1^, which further highlights the impact of a sidechain-free structure.^126^ By conducting *ex situ* GIWAXS measurements, it could be seen that the coherence lengths, which are reflective of the order in the film, increased upon electrochemical doping by 9% for **BBL**. In contrast, coherence lengths for **P-90** after doping reduced by 15%, showing a decrease in order in the film.^126^ This was also verified by examining the π-stacking distances, which is already shorter in **BBL** (3.51 Å, compared to 3.82 Å in **P-90**) and further contracts upon doping, which is likely beneficial for charge transport.^126^

However, as a result of the lack of sidechains, processing of BBL requires aggressive conditions, such as methanesulfonic acid. One particular area where **BBL** requires improvement is the response time, which is expectedly slow (∼1 s) on account of the limited diffusion of ions throughout the active layer.^7,8^

The concept of a fully fused polymer backbone was further investigated with the recent publication of **PgNaN** and **PgNgN** (Fig. 16), which builds on OTFT polymers **P3**, **P4** and **P5** (Fig. 11) by substituting the alkyl sidechains with glycol sidechains.^149^ Here, the materials were designed to achieve a near torsion-free π-conjugated backbone with an A–A configuration (Fig. 4). This electron deficient structure led to very high EA values of 4.28 and 4.35 eV respectively, and high performing OECT n-type mobilities of up to 6.50 ± 1.01 × 10^−3^ cm^2^ V^−1^ s^−1^ (Table 8), on account of the very well delocalised LUMO orbitals,^36^ surpassing the previous best performing OECT material, **BBL**, by an order of magnitude. Perhaps even more notably both **PgNaN** and **PgNgN** are synthesised *via* a metal-free aldol condensation polymerisation, an acid catalysed coupling absent of any toxic metals, common to most polymerisations, a major benefit for potential bioelectronic or sensing applications.^144^

The two polymers differ only by the composition of the sidechains, with **PgNgN** offering a fully glycolated backbone, whilst **PgNaN** displays a 50 : 50 ratio of alkyl to glycol sidechains. Yet this is enough to have a significant impact on performance, with the all glycol derivative showing a [μC*] value an order of magnitude lower than that of the mixed alkyl/glycol derivative. This is similar to the trend observed by the NDI series including **P-90** and **P-100**,^142^ again indicating that incorporation of some alkyl sidechains aids OECT performance, likely due to the ability of alkyl chains to modulate swelling of OECT materials.^143^ The use of an alkylated monomer, to form **PgNaN**, benefits both solubility and processability leading to an increase in polymer molecular weight.^149^ Therefore, to decisively state whether **PgNaN** is higher performing due to its incorporation of alkyl sidechains, polymers of the same molecular weight should be compared.

The fused structure of these rigid rod-like polymers prevents twisting due to the double bond which locks the conformation of connecting monomer units, minimising disorder and trapping sites, optimising charge transport along the backbone.^7,51^ Coupled with the highly electron-deficient acceptor–acceptor configuration these fused glycolated polymers are a promising new class of high performing OECT materials.

#### 3.2.3. Summary of OECT material design considerations

Material hydrophilicity facilitates aqueous swelling and subsequent ion transport into the active layer to increase the volumetric capacitance, and as such, common OECT materials incorporate glycol sidechains to introduce this property.^80,142^ However, empirical evidence shows that replacement of alkyl chains with glycol chains appears to reduce solubility in organic solvents used for polymerisations when compared to their alkyl counterparts, making high molecular weight glycolated polymers difficult to synthesise.^142^

Other design strategies for improving OECT performance including planarising the backbone utilising carbon–carbon double bonds to “lock” the conformation,^149^ increasing the electron deficiency of the repeat units,^81^ and replacing the traditional donor–acceptor moiety^142^ for an acceptor–acceptor backbone to deepen the LUMO and facilitate electron injection.^149^

## 4. n-Type organic thermoelectric devices

### 4.1. Assessing charge transport in n-type organic thermoelectric devices

**Fig. 17:**
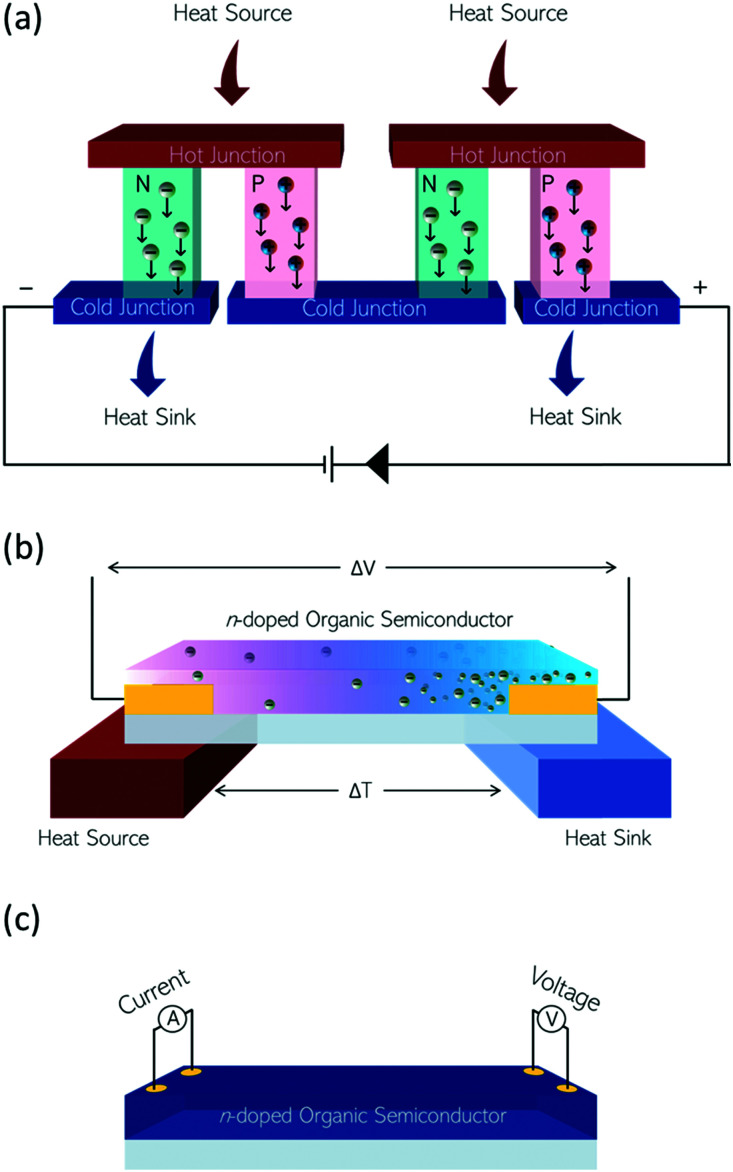
(a) Illustration of a thermoelectric generator employing both p-type and n-type materials (b) illustration of a setup for measuring the Seebeck coefficient in which the thermovoltage (Δ*V*) is measured across a thermal gradient (Δ*T*) (c) illustration of the setup for a Van der Pauw measurement in which Resistivity (the inverse of electrical conductivity) is calculated by measuring voltage and current in parallel across films of the organic semiconductor.

OTE generators convert thermal energy into power by applying a thermal gradient across OSCs. This gradient causes charge carriers to diffuse away from the heated side of the OTE material, thereby generating a potential difference across the material, known as a thermovoltage and given by the Seebeck coefficient (*S*), which is the ratio of voltage difference to temperature difference across the material. This Seebeck effect is the basis for power generation in thermoelectric (TE) generators. In a simple TE generator p- and n-type semiconductors are connected electrically in series and thermally in parallel (Fig. 17(a)). Criteria for a good OTE material are a high electrical conductivity combined with low thermal conductivity, to maintain the thermal gradient across the OTE material (typically, OSCs have low thermal conductivities of 0.1–1 W m^−1^ K^−1^).^66^ Whilst in OTFTs and OECTs the switching speed of the device and therefore the charge carrier mobility is critical in dictating performance, for OTEs, which are not required to transduce a signal, the electrical conductivity is more relevant. Electrical conductivity (*σ*) is proportional to both the charge carrier mobility, and the charge carrier density (*n*) as in eqn (5), where *q* is the elementary charge.

5 *σ* = *qnμ*

Critically, in contrast to *σ*, *S* is inversely proportional to charge carrier density.^163^ For this reason to realise the best performance of an OSC for TE generators *n* must be optimised to maximise the power factor (PF), which combines *σ* and *S* (eqn (6)). For OTE materials this has often been achieved through the design of polymers compatible with n-type dopants, as will be discussed in the next sections. The majority charge carrier determines the sign of *S*, positive for p-type OTE materials and negative for n-type OTE materials. In order to compare p- and n-types, a *S*^2^ value is calculated so that all materials can be benchmarked using a positive term to determine PF.^164^

6 PF = *S*^2^*σ*

Materials for TE generators are compared using the figure of merit *ZT*(7), where *T* is temperature and *Z* combines the PF and thermal conductivity (*κ*). This dimensionless figure of merit applies to both n- and p-type materials and the most efficient TE generators are comprised of p- and n-type materials with similar *ZT* values.^165,166^ Practically, measuring the thermal conductivity of OSC films is limited and challenging. Instead, the performance of materials is often compared using the PF.7
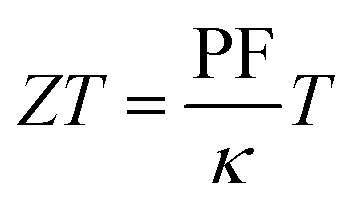
Whereas, in OTFTs and OECTs the performance of new OSC polymers is directly measured in the device, for OTE materials, the Seebeck coefficient and electrical conductivity are typically measured separately Fig. 17(b) and (c). A variety of methods are used to measure the bulk electrical conductivity of n-type OSC films, including the Van der Pauw method and voltage-source two-point conductivity.^70,167^ Conducting polymers and high mobility OSCs which can be doped to maximise their PF value are two classes of OTE materials that have been investigated.

### 4.2. Applications and limitations of n-type dopants

To optimise the power factor (6) and thereby *ZT*(7) of OTE materials charge carrier density can be optimised *via* n-doping, transferring electrons from the HOMO of the dopant into the LUMO of the OSC. Upon doping, (bi)polarons with differing delocalisation lengths are generated. Polymers with a D–A motif generally exhibit a more localised polaron, confined by the discrete regions of orbital density, which can potentially limit charge transport,^168^ when compared to the A–A motif, where the LUMO tends to be more delocalised along the backbone.

Limitations of n-type dopants include their redox stability and the immiscibility of the oxidised species with the OSC as well as the adverse impact on OSC morphology by disrupting packing.^70,169,170^ The inherent instability of n-dopants has limited the development of n-type OTE materials compared with their p-type counterparts. The very shallow dopant HOMO levels required to n-type dope OSCs mean air stable n-dopants are rare, as possessing such a low IP increases the dopants susceptibility to oxidation by ambient species.^45^ For efficient n-type doping of OSCs, a deep OSC LUMO level below −4.0 eV is required.^66^

**Fig. 18:**
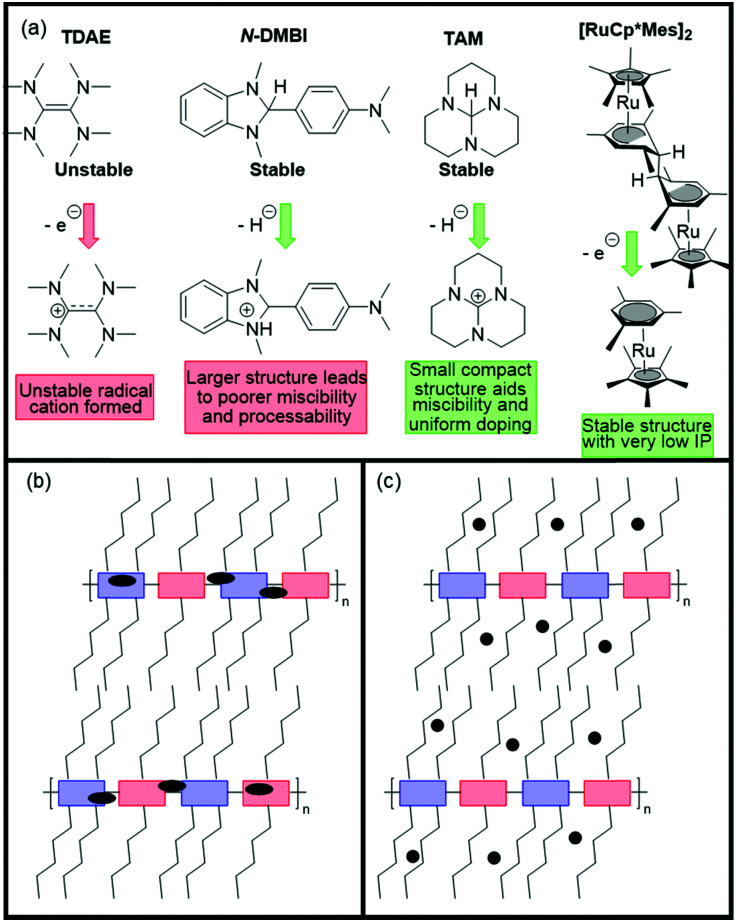
(a) Four n-type dopants commonly used in OTE applications, their neutral ambient stabilities and the ability of the cations to assist in the doping process, (b) a schematic representation of the doping of **FBDPPV** with **N-DMBI** and with (c) **TAM**.^170^

There is a slim selection of n-dopants for application in OTEs, including tetrakis(dimethylamino)ethylene (**TDAE**),^171^ 4-(1,3-dimethyl-2,3-dihydro-1*H*-benzoimidazol-2-yl)-*N*,*N*-dimethylaniline (**N-DMBI**)^172^ and triaminomethane (**TAM**) (Fig. 18(a)).^170^**TDAE** is not air stable, and therefore has limited applications in OTEs. **N-DMBI** is one of the most commonly used and best understood n-type dopants for OTE materials, likely due to the ambient stability of the native species.^66,172,173^ Stability of **N-DMBI** and **TAM** as n-type dopant precursors originate from the mechanism of doping, which is *via* a latent hydride (formed *in situ*) or an initial hydrogen removal, rather than simply donating an electron, meaning requirements of the n-type dopant to have a low IP are alleviated.^154,170,172^ When using **N-DMBI**, the resulting cation has exhibited poor miscibility with typical alkyl substituted n-type OSCs, one rationale for investigating more polar sidechains.^174^

**TAM** is the state-of-the-art n-dopant as it maintains the excellent n-doping abilities of **N-DMBI**, whilst improving its miscibility with alkylated polymers, meaning n-type OTEs can benefit from the catalogue of pre-existing polymers developed for n-type OTFT applications. **TAM** enables uniform doping, with minimal effect on the π–π stacking of polymer backbones, as demonstrated with n-type polymer **FBDPPV** (Fig. 20).^170^ This occurs as the TAM^+^ cation has a small molecular volume and remains in the sidechain region due to a weak affinity with the π-backbones and a stronger affinity with the polymer sidechains, due to compatibility with its own cyclic alkyl sidechains.^167^ Conversely **N-DMBI** typically moves into the π-backbone region to avoid incompatible polarities between the dopant molecule and the alkyl sidechains, as predicted by statistical analysis of equilibrium-state counterion-backbone distances.^170^ This can be explained by DFT calculations, which predict that TAM^+^ has a stronger affinity with alkyl sidechains due to the respective polarizabilities of the dopant and the sidechain region, thus enabling TAM^+^ to pack more tightly than **N-DMBI** during doping (Fig. 18(b) and (c)).

Another approach in the direction of air-stable n-type dopants is the use of organometallic dimers with high reducing abilities.^175^ These are able to cleave *in situ*, which is followed by a rapid electron transfer to the OSC. The n-dopant, (pentamethylcyclopentadienyl)(1,3,5-trimethylbenzene)ruthenium ([RuCp*Mes]2), is particularly impressive as it has a very shallow HOMO, so is able to dope polymers with LUMOs as shallow as −2.8 eV (Fig. 18(a)).^176^

Thermal stability of the doped n-type OSC is key, especially for thermoelectric applications. Films of OSCs doped with **N-DMBI** exhibited poor thermal stability, with the observed loss of *σ*_max_ attributed to de-doping.^177^ Good thermal stability was observed in another study sequentially doping with **N-DMBI**, that is doping the thin film rather than solution of the OSC.^178^ Improved thermal stability when using **N-DMBI** has also been observed when there is good miscibility between the polymeric OSC and dopant, for example when polar side chains are used.^70^ Adequate miscibility between the polymer and dopant enables a uniform distribution of dopant molecules within the polymer matrix, limiting aggregation and ultimately doping a greater proportion of the polymer, enhancing TE performance.^179^ This was corroborated by a recent study comparing the doping efficiency and resultant thermoelectric performance of the dimeric **(N-DMBI)2** dopant (which forms the same N-DMBI^+^ cation as **N-DMBI**, without involving a hydrogen atom or hydride transfer) against the (RuCp*mes)2 dopant (Fig. 18(a)) for **FBDPPV** containing OTE devices.^180^ These results suggest that, at least for the doping of ordered polymers, molecular dopants should be designed to have a more planar shape to minimise perturbation of ordered microstructures and to facilitate efficient electron-transfer reaction pathways.

It is clear that the dopant and its interactions with the n-type OSC are vital to maintaining a stable value of *σ*_max_ under prolonged thermal stress. This discussion highlights that the choice of dopant can be considered equally important as the n-type OSC material itself, making comparison between new materials difficult and rational design of n-type OSCs for OTEs is restricted by and often relies upon the available dopants.

### 4.3. Organic thermoelectric materials

Organic materials are promising TE candidates owing to their intrinsically low thermal conductivity, diverse molecular design and solution processability. The previously discussed instabilities relating to n-dopants (Section 4.2) have limited the development of high performing OTE materials, with n-type OTE performance lagging behind their p-type counterparts. Recently, however, advancements have been made to address the poor n-doping efficiency and TE performance of n-type polymers, including the synthetic modification of traditional n-type polymers,^66,181^ the fabrication of novel n-type conjugated polymers,^58,182^ and the development of more efficient n-type dopants and doping processes.^183,184^ As is the case for the OSCs employed in n-type OTFTs and OECTs, again for high n-type OTE performance deepening the LUMO energy level (Fig. 4) and consideration of the electronic structure of the material is required. Similarly to n-type OECTs, the introduction of an extrinsic charged component into the OSC, in the case of OTEs an n-type dopant, means again for this application often polar side chains are exploited to aid the miscibility of the dopant and OSC. In order to gain some insight into the structure–property relationships of these emerging n-type OTE materials, we have again grouped them into five general categories: NDI-based derivatives,^171,174,181^ polylactam/lactone derivatives,^66,185^ NDTI-based copolymers,^58,107^ DPP-based copolymers^182,186^ and fully fused A–A derivatives.^63,171^

#### 4.3.1. Naphthalenediimide (NDI) derivatives

**Fig. 19:**
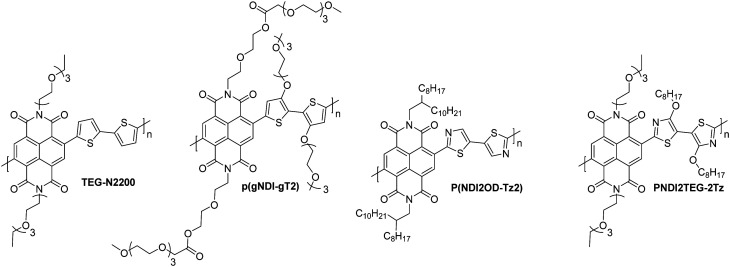
Chemical structures of selected n-type thermoelectric NDI polymer derivatives, including published synonyms.

Early investigations doping **N2200** (with **N-DMBI**; Fig. 18), showed that only 1% of the dopant was active, indicating that poor miscibility between **N2200** and **N-DMBI** greatly hindered the doping efficiency, resulting in poor TE performance.^187^ Molecular dynamic simulations showed that replacing the non-polar hydrophobic alkyl chains with hydrophilic polar triethylene glycol (TEG) sidechains enabled more uniform dispersion of the dopant into the polymer matrix. Indeed, the glycolated **N2200** (**TEG-N2200**) (Fig. 19) upon doping with **N-DMBI** exhibits an enhanced *σ*_max_, *ca.* 200 times greater than that of **N2200**, at 0.17 S cm^−1^ (Table 9).^181^ These findings are supported by **p(gNDI-gT2)**, which introduces TEG side chains to the thiophene unit as well as extended polar side chains on the NDI unit compared with **N2200**. This results in a further increase in TE performance with a *σ*_max_ almost two times that of **TEG-N2200**, at 0.30 S cm^−1^, due to enhanced doping efficiency on account of the increased glycol content (Table 9).^174^ Notably, upon doping, the air stability of **p(gNDI-gT2)** was greatly enhanced over the pristine undoped state, demonstrating the potential importance of OEG sidechains for the development of stable n-type TE materials.^188^

**Fig. 20:**
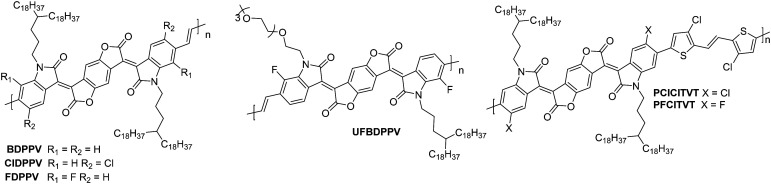
Chemical structures of selected n-type thermoelectric polylactam/lactone derivatives, including published synonyms.

**Table 9:** Published polymer synonyms, electron affinity (EA), number and weight average molecular weights (*M*_n_/*M*_w_), thermoelectric dopant, electrical conductivity (*σ*_max_), power factor (PF) and associated reference for selected NDI polymer derivatives

Polymer	EA^a^ (eV)	*M*_n_/*M*_w_ (kDa)	Dopant	*σ*_max_ (S cm^−1^)	PF (μW m^−1^ K^−2^)	Ref.
**N2200**	3.90	Not reported	*N*-DMBI	0.008	0.6	187
**TEG-N2200**	3.96	3.8/4.6^b^	*N*-DMBI	0.2	0.4	181
**p(gNDI-gT2)**	4.10	8.8^c^	*N*-DMBI	0.3	0.4	174
**P(NDI2OD-Tz2)**	4.10	32.2/54.7^b^	TDAE	0.1	1.5	171
**PNDI2TEG-2Tz**	4.26	26.2/29.7^b^	*N*-DMBI	1.8	4.5	189

a EA is an estimation of the LUMO, although neglects the electron binding energy.

b These molecular weight values were determined by GPC.

c This molecular weight value was determined by MALDI-TOF.

An alternative approach is to replace the bithiophene (T2) unit of **N2200** with a bithiazole (Tz2) moiety, affording **P(NDI2OD-Tz2)** (Fig. 19), a polymer with improved π–π stacking due to reduced intrachain steric demands in Tz2 *versus* T2, leading to greater backbone planarity.^171^ The increased electron-deficient character of Tz2 increases the EA and decreases the D–A character (Fig. 4), resulting in a *σ*_max_ of 0.1 S cm^−1^ (Table 9), rising by two orders of magnitude compared to **N2200** also doped with **TDAE** (Fig. 18; 0.003 S cm^−1^).^159,187^

#### 4.3.2. Polylactam/lactone derivatives

Glycolation is again utilised in the material **PNDI2TEG-2Tz**, with TEG sidechains on the NDI unit, coupled with an alkylated bithiazole co-monomer (Fig. 19). Compared to **N2200**, due to the decreased π–π stacking distance between polymer units imparted by the planar backbone configuration, a narrower distribution of density of states (DOS) is achieved, which was measured using a direct electrochemical method.^189^ As both *σ*_max_ and the Seebeck coefficient are closely related to the DOS distribution, the contraction associated with changing from T2 to Tz2, resulted in a marked increase in TE device performance, **PNDI2TEG-2Tz** doped with **N-DMBI** recorded a *σ*_max_ of 1.8 S cm^−1^ and a PF of 4.5 μW m^−1^ K^−2^ (Table 9).^189^

The benzodifurandione-based polymer (**BDPPV**) exhibits a high EA value of 4.0 eV and forms part of a series with **ClBDPPV** and **FBDPPV** (Fig. 20). These polymers introduce halogen atoms, which increase the EA by 0.3 and 0.17 eV respectively, improving doping efficiency and more than doubling electron mobility, compared to **BDPPV** (Table 9).^66^ This can be attributed to the electron-withdrawing and planarizing effects of including pendant fluorine atoms, which act as a conformational lock through intrachain non-covalent short contacts to minimise the dihedral angle about the double bond between the lactone and isatin moiety. As a result, upon doping with **N-DMBI** both **ClBDPPV** and **FBDPPV** show increased *σ*_max_ and PF compared to unhalogenated **BDPPV**. Further improved PF values of 51 μW m^−1^ K^−2^ were reported for **FBDPPV** by incorporating the newly-developed n-dopant **TAM** (Fig. 18).^68^ The original **BDPPV** polymer backbone was further modified through glycolation, replacing one alkyl chain with an OEG chain to furnish **UFBDPPV** (Fig. 20). Upon doping with **TAM**, a very impressive OTE performance was reported, with *σ*_max_ of 22.5 S cm^−1^ and a PF of 80 μW m^−1^ K^−2^.^167^ The increased *σ*_max_ of **UFBDPPV** is due to the highly miscible **TAM** cations which do not disturb the polymer microstructure and enable an efficient interchain charge transport in the conductive films. The asymmetric sidechain distribution of **UFBDPPV** allows for this amphipathic polymer to benefit from both excellent dopant-polymer miscibility, whereby dopant molecules are confined to the hydrophilic sidechain region, whilst retaining good π–π packing thus increasing doping efficiency and *σ*_max_.^190^ The benefit of including OEG-based sidechains for positive polymer-dopant interactions has been corroborated through multiple studies and has been proven as an effective method for increasing OTE performance.^174,180,181,191^

The inherent instability of n-type materials drove interest in blocking the polymer backbone from contact with oxygen and water during device operation.^192^ As such, building upon the previous BDPPV-based polymers, a difluoro- and dichloro-substituted form of the electron-deficient BDOPV unit were coupled with the relatively weak donor moiety dichlorodithienylethene (ClTVT), resulting in two D–A BDOPV-based polymers, **PClClTVT** and **PFClTVT**, designed to minimise backbone contact with ambient species (Fig. 20).^67^ Despite the two polymers sharing an identical backbone, the replacement of chlorine with fluorine atoms on the electron-deficient BDOPV unit has a remarkable influence on the *σ*_max_, which is rationalised with the same explanation as for the **BDPPV** polymer series. Indeed, *σ*_max_ of **PClClTVT** is 16.1 S cm^−1^, which is less than half that of **PFClTVT** (38.3 S cm^−1^), furthermore the PF of **PFClTVT** reached 22.7 μW m^−1^ K^−2^, three times that of **PFClTVT** (Table 10).^67^ Once again, the drastically improved *σ*_max_ and resultant TE performance of both these materials, particularly **PFClTVT**, are dominated by the charge carrier mobility, following eqn (5). It should also be noted that the authors reported that **PClClTVT**, doped with 50 mol% **N-DMBI**, retained a *σ*_max_ value of 4.9 S cm^−1^ after storing in air for 222 days, a very considerable stability for an n-doped polymer stored in air.^67^

**Table 10:** Published polymer synonyms, electron affinity (EA), number and weight average molecular weights (*M*_n_/*M*_w_), thermoelectric dopant, electrical conductivity (*σ*_max_), power factor (PF) and associated reference for selected polylactam/lactone derivatives

Polymer	EA^a^ (eV)	*M*_n_/*M*_w_*M*_n_/*M*_w_^b^ (kDa)	Dopant	*σ*_max_ (S cm^−1^)	PF (μW m^−1^ K^−2^)	Ref.
**BDPPV**	4.01	41.8/99.9	*N*-DMBI	0.3	1.6	66
**ClBDPPV**	4.30	38.6/97.3	*N*-DMBI	7.0	16.5	66
**FBDPPV**	4.17	42.9/101.2	*N*-DMBI	14.0	28.0	66
**UFBDPPV**	4.13	34.5/122.8	TAM	22.5	80.0	167
**PClClTVT**	4.00	58.6/130.6	*N*-DMBI	16.1	7.6	67
**PFClTVT**	4.03	39.4/140.6	*N*-DMBI	38.3	22.7	67

a EA is an estimation of the LUMO, although neglects the electron binding energy.

b Molecular weight values were determined by GPC.

#### 4.3.3. Naphthodithiophenediimide-based (NDTI) copolymers

**Fig. 21:**
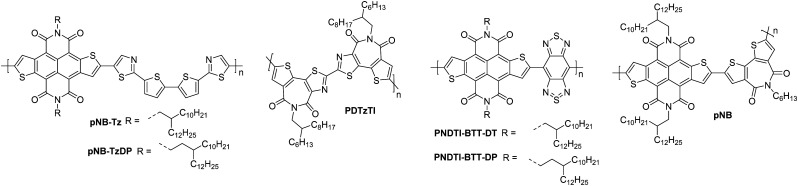
Chemical structures of selected n-type thermoelectric NDTI polymer derivatives, including published synonyms.

The selection of NDTI polymers in Fig. 21 utilise backbone engineering to influence OTE performance.^107^ The first polymer, **pNB** (Fig. 21), employs the A–A motif, by copolymerising NDTI with an electron-deficient bithiopheneimide (BTI) unit. The flexible backbone configuration limited electron transport and the resultant TE performance. The BTI unit was then replaced with an extended thiazole moiety, leading to a pseudo straight-line rigid conformation, namely **pNB-Tz** and **pNB-TzDP**, which only differ by a single carbon atom within the branching chains (Fig. 21).^107^ The increased crystallinity, originating from closer π–π and lamellar stacking of polymer backbones, is beneficial to charge transport. These modifications led to *σ*_max_ of 11.6 S cm^−1^ and a PF of 53.4 μW m^−1^ K^−2^ for **pNB-TzDP**, a two-order of magnitude increase over the performance of **pNB** (Table 11).^107^

**Table 11:** Published polymer synonyms, electron affinity (EA), number and weight average molecular weights (*M*_n_/*M*_w_), thermoelectric dopant, electrical conductivity (*σ*_max_), power factor (PF) and associated reference for selected NDTI polymer derivatives

Polymer	EA^a^ (eV)	*M*_n_/*M*_w_^b^ (kDa)	Dopant	*σ*_max_ (S cm^−1^)	PF (μW m^−1^ K^−2^)	Ref.
**pNB**	4.20	11.5/18.4	*N*-DMBI	0.01	0.3	107
**pNB-Tz**	4.22	11.6/22.0	*N*-DMBI	0.9	9.9	107
**pNB-TzDP**	4.22	15.4/35.4	*N*-DMBI	11.6	53.4	107
**PDTzTI**	3.80	7.2/7.7	TDAE	4.6	7.6	184
**PNDTI-BTT-DT**	4.40	17.6/83.4	*N*-DMBI	0.12	0.6	58
**PNDTI-BTT-DP**	4.40	20.5/51.9	*N*-DMBI	5.0	14.2	58

a EA is an estimation of the LUMO, although neglects the electron binding energy.

b Molecular weight values were determined by GPC.

Although the BTI unit imparts a wave-line backbone for the **pNB** polymer, as predicted by DFT calculations,^107^ a BTI homopolymer, **PDTzTI**, (Fig. 21) exhibits a near coplanar backbone with enhanced crystallinity.^89^ Moreover, closer π–π stacking and A–A character further improved charge carrier generation and transportation compared to **pNB**. As a result, **PDTzTI** achieved a notable *σ*_max_ of 4.6 S cm^−1^ and a PF of 7.6 μW m^−1^ K^−2^ (Table 11).^184^

#### 4.3.4. Diketopyrrolopyrrole-based copolymers

**Fig. 22:**
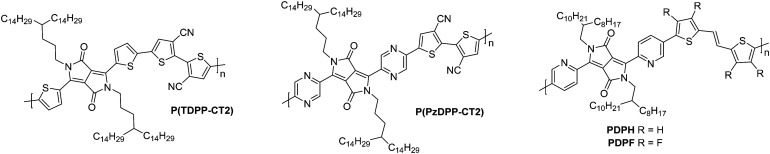
Chemical structures of selected n-type thermoelectric DPP polymer derivatives, including published synonyms.

Another promising class of n-type TE materials are DPP-based D–A copolymers, with high crystallinity and *μ*_e_ over 5 cm^2^ V^−1^ s^−1^ reported.^193,194^ A recent example of a DPP-based copolymer presented a DPP unit flanked with either thiophene or pyrazine, before copolymerising with 2,2′-dicyanobithiophene affording **P(TDPP-CT2)** and **P(PzDPP-CT2)**, respectively (Fig. 22).^182^ The planar backbone improves crystallinity and reduces π–π stacking distances, which increases charge carrier mobility for **P(PzDPP-CT2)** over **P(TDPP-CT2)**. This is mirrored by the TE device performance, whereby **P(PzDPP-CT2)**, with *σ*_max_ of up to 8.4 S cm^−1^ and a PF up to 57.3 μW m^−1^ K^−2^, is among the highest reported for n-type materials (Table 12).^182^ Given the earlier discussion regarding the inclusion of polar sidechains for improving dopant miscibility, it should be noted that there currently aren’t any examples of this for DPP-based polymers.

**Table 12:** Published polymer synonyms, electron affinity (EA), number and weight average molecular weights (*M*_n_/*M*_w_), thermoelectric dopant, electrical conductivity (*σ*_max_), power factor (PF) and associated reference for selected DPP polymer derivatives

Polymer	EA^a^ (eV)	*M*_n_/*M*_w_^b^ (kDa)	Dopant	*σ*_max_ (S cm^−1^)	PF (μW m^−1^ K^−2^)	Ref.
**P(TDPP-CT2)**	3.70	34.0/73.0	*N*-DMBI	0.4	9.3	182
**P(PzDPP-CT2)**	4.03	28.5/82.8	*N*-DMBI	8.4	57.3	182
**PDPH**	3.93	30.9/75.4	*N*-DMBI	0.001	0.0005	186
**PDPF**	4.11	29.9/75.6	*N*-DMBI	1.3	4.7	186

a EA is an estimation of the LUMO, although neglects the electron binding energy.

b Molecular weight values were determined by GPC.

#### 4.3.5. Fully fused acceptor–acceptor derivatives

On top of **BBL**'s desirable coplanar torsion free backbone, good charge transport properties and lack of solubilising groups discussed in previous sections, DFT calculations have demonstrated that spin density distributions along the polymer backbone extend over three repeat units, confirming extended polaron delocalisation in ladder type A–A polymers compared to their D–A counterparts where polarons tend to be more localised on the acceptor unit.^51^**TDAE** was chosen as the n-dopant, as it has previously been optimised with one of the best performing p-type thermoelectric materials, poly(3,4-ethylenedioxythiophene) (**PEDOT**), and consistency of dopant choice across both p- and n-type components of an OTE device may provide ease of device assembly.^195^ Indeed, **BBL** devices doped with **TDAE** exhibit *σ*_max_ of 2.4 S cm^−1^ and a PF of 0.43 μW m^−1^ K^−2^,^159^ greatly outperforming “structurally distorted” D–A polymers such as **P(NDI2OD-Tz2)** and other NDI derivatives (Table 13).

**Table 13:** Published polymer synonyms, electron affinity (EA), number and weight average molecular weights (*M*_n_/*M*_w_), thermoelectric dopant, electrical conductivity (*σ*_max_), power factor (PF) and associated reference for selected fully fused polymer derivatives

Polymer	EA^a^ (eV)	*M*_n_/*M*_w_^b^ (kDa)	Dopant	*σ*_max_ (S cm^−1^)	PF (μW m^−1^ K^−2^)	Ref.
**BBL**	4.00	Not reported	TDAE	2.4	0.43	159
**N–N**	3.94	80/215	*N*-DMBI	0.65	3.2	63
**A–N**	3.83	139/480	*N*-DMBI	0.26	1.6	63
**A–A**	3.72	51/162	*N*-DMBI	0.018	0.25	63
**LPPV-1**	4.49	15.8/42.2	TAM	4.0	34.8	196

a EA is an estimation of the LUMO, although neglects the electron binding energy.

b Molecular weight values were determined by GPC.

**Fig. 23:**
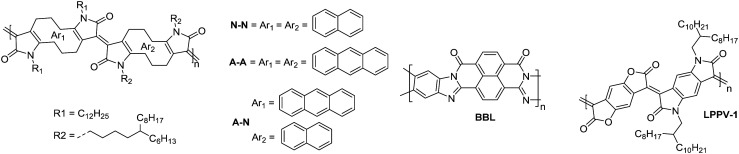
Chemical structures of selected n-type thermoelectric fully fused polymer derivatives, including published synonyms.

In a series of three fully fused polylactams (Fig. 23), reducing the central acene core size resulted in progressively increasing EA values (Fig. 4), leading to an increasingly more favourable and efficient n-type doping with **N-DMBI**. Specifically, reducing the central lactam core from two anthracenes (**A–A**) to mixed anthracene–naphthalene (**A–N**) and finally to two naphthalene cores (**N–N**) yielded a more delocalised electron polaron and a larger EA of 3.94 eV for the N–N polymer (Fig. 23). The benefits of contracting the acene core were further substantiated by the increased TE performance, with **N–N** displaying the highest *σ*_max_ (0.65 S cm^−1^) and PF (3.2 μW m^−1^ K^−2^) of the series.^63^

In **LPPV-1** (Fig. 23),^196^ an isoindigo derivative with a coplanar rigid backbone, the central fused carbon–carbon double bond in addition to the intramolecular non-covalent short contacts between the phenyl hydrogen and adjacent oxygen atom renders the backbone near torsion free. The acceptor–acceptor character, electron-deficient lactone core, and the incorporation of electron-withdrawing moieties within the backbone led to an extremely high EA of 4.5 eV, desirable for n-doping (Fig. 4). Indeed, upon doping with **N-DMBI**, *σ*_max_ of 1.1 S cm^−1^ and a PF of 1.96 μW m^−1^ K^−2^ were achieved. Akin to **UFBDPPV** (Table 10), the TE performance of **LPPV-1** was also improved drastically upon doping with **TAM**, reaching *σ*_max_ of up to 4.0 S cm^−1^ and a PF of 34.8 μW m^−1^ K^−2^ (Table 13).^167^ This investigation suggests that, without sacrificing Seebeck coefficients, high conductivities can be realised with precise regulation of the interaction between the cations and the host. Ultimately both the intrinsic polymer performance parameters (planarity, LUMO level, mobility) and the interaction between polymer and dopant must be optimised to maximise OTE performance.

## 5. Summary

This review has summarised the various design strategies employed to maximise performance of electron transporting OSC materials for use in OTFT, OECT and OTE device applications as well as figures of merit used to measure performance. The inherent limitations associated with n-type materials are discussed, which arise from the operational instability in ambient conditions of a material with a LUMO shallower than −4.0 eV.^66^ This value is calculated by examining the reduction potentials of water and oxygen, and accounting for overpotentials of these reactions.^47^ n-Type instability can also be understood by examining the extent to which the LUMO is delocalised across the entire polymer backbone; by improving this, electrons move freely throughout the length of the backbone, rather than becoming localised on the highly electron deficient component, making it more susceptible to oxidation.^40^ Instabilities can also arise in the presence of photogenerated singlet oxygen, which can cause photobleaching of OSC materials.^48^ Consideration should therefore be given to both the material design to lower the LUMO below −4.0 eV and improve delocalisation of the LUMO across the entire polymer backbone (through improved planarity and an acceptor–acceptor configuration), in addition to the device architecture and processing conditions to maximise charge transport.

This review also addresses the challenges in extracting comparable data, including variations in how measurements are conducted, differing device architectures and lack of clarity or overinflation when reporting numbers.

Despite the variance in device architectures, the improved structure–performance properties published by multiple research groups tend to follow similar trends which include; judicious use of electron deficient units to deepen LUMO energy levels,^60,66,85^ extending conjugation,^36,58,59,149^ maximising molecular weights,^14,15,21^ and altering polymer backbone electronic configuration, for example by employing an acceptor–acceptor motif.^64,197,198^ The material systems represented throughout, are evidence of the viability of invoking multiple different design principles to improve the performance of n-type materials towards three different fields of organic electronics, namely OTFTs, OECTs and OTEs. The recent acceleration of research and development has been rewarded with the performance of n-type materials that are beginning to approach performance seen in their p-type counterparts.

Potential directions for future work include employing strategies to maximise capacitance without compromising processability (as seen for **BBL**),^7^ methods to increase the molecular weight of glycolated polymers to improve mobility, and finding novel methods for improving stability of both n-type materials and n-dopants.^170^

The area of organic n-type materials continues to expand and with promising molecular design concepts to explore, the future of the field is extremely promising.

## Conflicts of interest

There are no conflicts to declare.

## References

[cit1] Paquin F., Latini G., Sakowicz M., Karsenti P. L., Wang L., Beljonne D., Stingelin N., Silva C. (2011). Phys. Rev. Lett..

[cit2] Yamashita Y. (2009). Sci. Technol. Adv. Mater..

[cit3] Sze S. M. and Ng K. K., Physics of Semiconductor Devices, Wiley, Hoboken, New Jersey, 3rd edn, 2006

[cit4] Zaumseil J., Sirringhaus H. (2007). Chem. Rev..

[cit5] Skotheim T. A. and Reynolds J. R., Handbook of Conducting Polymers. Conjugated Polymers: Processing and Applications, CRC Press, Boca Raton, Florida, 3rd edn, 2007

[cit6] Zhan C. G., Nichols J. A., Dixon D. A. (2003). J. Phys. Chem. A.

[cit7] Sun H., Vagin M., Wang S., Crispin X., Forchheimer R., Berggren M., Fabiano S. (2018). Adv. Mater..

[cit8] Sun H., Gerasimov J., Berggren M., Fabiano S. (2018). J. Mater. Chem. C.

[cit9] Takeda Y., Hayasaka K., Shiwaku R., Yokosawa K., Shiba T., Mamada M., Kumaki D., Fukuda K., Tokito S. (2016). Sci. Rep..

[cit10] Li H., Kim F. S., Ren G., Jenekhe S. A. (2013). J. Am. Chem. Soc..

[cit11] Facchetti A. (2011). Chem. Mater..

[cit12] Fritz S. E., Martin S. M., Frisbie C. D., Ward M. D., Toney M. F. (2004). J. Am. Chem. Soc..

[cit13] Horowitz G. (1998). Adv. Mater..

[cit14] Coropceanu V., Cornil J., da Silva Filho D. A., Olivier Y., Silbey R., Brédas J. L. (2007). Chem. Rev..

[cit15] Zen A., Pflaum J., Hirschmann S., Zhuang W., Jaiser F., Asawapirom U., Rabe J. P., Scherf U., Neher D. (2004). Adv. Funct. Mater..

[cit16] Pasveer W. F., Cottaar J., Tanase C., Coehoorn R., Bobbert P. A., Blom P. W. M., De Leeuw M., Michels M. A. J. (2005). Phys. Rev. Lett..

[cit17] Roichman Y., Tessler N. (2003). Synth. Met..

[cit18] Tanase C., Meijer E. J., Blom P. W. M., de Leeuw D. M. (2003). Phys. Rev. Lett..

[cit19] Kim D. H., Lee J., Il Park J., Chung J. W., Lee W. H., Giri G., Yoo B., Koo B., Kim J. Y., Jin Y. W., Cho K., Lee B. L., Lee S. (2011). Adv. Funct. Mater..

[cit20] Trefz D., Ruff A., Tkachov R., Wieland M., Goll M., Kiriy A., Ludwigs S. (2015). J. Phys. Chem. C.

[cit21] Nahid M. M., Matsidik R., Welford A., Gann E., Thomsen L., Sommer M., McNeill C. R. (2017). Adv. Funct. Mater..

[cit22] Shin Y. H., Komber H., Caiola D., Cassinelli M., Sun H., Stegerer D., Schreiter M., Horatz K., Lissel F., Jiao X., McNeill C. R., Cimò S., Bertarelli C., Fabiano S., Caironi M., Sommer M. (2020). Macromolecules.

[cit23] Noriega R., Rivnay J., Vandewal K., Koch F. P. V., Stingelin N., Smith P., Toney M. F., Salleo A. (2013). Nat. Mater..

[cit24] Gu K., Onorato J. W., Luscombe C. K., Loo Y. L. (2020). Adv. Electron. Mater..

[cit25] Fratini S., Nikolka M., Salleo A., Schweicher G., Sirringhaus H. (2020). Nat. Mater..

[cit26] Sirringhaus H. (2005). Adv. Mater..

[cit27] Takacs C. J., Treat N. D., Krämer S., Chen Z., Facchetti A., Chabinyc M. L., Heeger A. J. (2013). Nano Lett..

[cit28] Chen Z., Zheng Y., Yan H., Facchetti A. (2009). J. Am. Chem. Soc..

[cit29] Pandey M., Kumari N., Nagamatsu S., Pandey S. S. (2019). J. Mater. Chem. C.

[cit30] Shi J., Li Y., Jia M., Xu L., Wang H. (2011). J. Mater. Chem..

[cit31] Fornari R. P., Rowe P., Padula D., Troisi A. (2017). J. Chem. Theory Comput..

[cit32] McCulloch I., Heeney M., Bailey C., Genevicius K., MacDonald I., Shkunov M., Sparrowe D., Tierney S., Wagner R., Zhang W., Chabinyc M. L., Kline R. J., McGehee M. D., Toney M. F. (2006). Nat. Mater..

[cit33] Venkateshvaran D., Nikolka M., Sadhanala A., Lemaur V., Zelazny M., Kepa M., Hurhangee M., Kronemeijer A. J., Pecunia V., Nasrallah I., Romanov I., Broch K., McCulloch I., Emin D., Olivier Y., Cornil J., Beljonne D., Sirringhaus H. (2014). Nature.

[cit34] Wang X.-Y., Lin H.-R., Lei T., Yang D.-C., Zhuang F.-D., Wang J.-Y., Yuan S.-C., Pei J. (2013). Angew. Chem..

[cit35] Okamoto T., Suzuki T., Tanaka H., Hashizume D., Matsuo Y. (2012). Chem. – Asian J..

[cit36] Onwubiko A., Yue W., Jellett C., Xiao M., Chen H. Y., Ravva M. K., Hanifi D. A., Knall A. C., Purushothaman B., Nikolka M., Flores J. C., Salleo A., Bredas J. L., Sirringhaus H., Hayoz P., McCulloch I. (2018). Nat. Commun..

[cit37] Fu C., Beldon P. J., Perepichka D. F. (2017). Chem. Mater..

[cit38] Statz M., Venkateshvaran D., Jiao X., Schott S., McNeill C. R., Emin D., Sirringhaus H., Di Pietro R. (2018). Commun. Phys..

[cit39] Rivnay J., Inal S., Salleo A., Berggren M., Malliaras G. G. (2018). Nat. Rev. Mater..

[cit40] Chua L., Zaumseil J., Chang J., Ou E. C., Ho P., Sirringhaus H., Friend R. H. (2005). Nature.

[cit41] Liu C., Li G., Di Pietro R., Huang J., Noh Y.-Y., Liu X., Minari T. (2017). Phys. Rev. Appl..

[cit42] Upadhyaya M., Boyle C. J., Venkataraman D., Aksamija Z. (2019). Sci. Rep..

[cit43] Fratini S., Nikolka M., Salleo A., Schweicher G., Sirringhaus H. (2020). Nat. Mater..

[cit44] Brixi S., Melville O. A., Mirka B., He Y., Hendsbee A. D., Meng H., Li Y., Lessard B. H. (2020). Sci. Rep..

[cit45] Jones B. A., Facchetti A., Wasielewski M. R., Marks T. J. (2007). J. Am. Chem. Soc..

[cit46] Quinn J. T. E., Zhu J., Li X., Wang J., Li Y. (2017). J. Mater. Chem. C.

[cit47] De Leeuw D. M., Simenon M. M. J., Brown A. R., Einerhand R. E. F. (1997). Synth. Met..

[cit48] Sudakov I., Van Landeghem M., Lenaerts R., Maes W., Van Doorslaer S., Goovaerts E. (2020). Adv. Energy Mater..

[cit49] Zhan X., Facchetti A., Barlow S., Marks T. J., Ratner M. A., Wasielewski M. R., Marder S. R. (2011). Adv. Mater..

[cit50] Usta H., Risko C., Wang Z., Huang H., Deliomeroglu M. K., Zhukhovitskiy A., Facchetti A., Marks T. J. (2009). J. Am. Chem. Soc..

[cit51] Babel A., Jenekhe S. A. (2003). J. Am. Chem. Soc..

[cit52] Kim R., Amegadze P. S. K., Kang I., Yun H.-J., Noh Y.-Y., Kwon S.-K., Kim Y.-H. (2013). Adv. Funct. Mater..

[cit53] Kang B., Kim R., Lee S. B., Kwon S.-K., Kim Y.-H., Cho K. (2016). J. Am. Chem. Soc..

[cit54] Roncali J. (2007). Macromol. Rapid Commun..

[cit55] Sommer M. (2014). J. Mater. Chem. C.

[cit56] Nielsen C. B., Turbiez M., McCulloch I. (2013). Adv. Mater..

[cit57] Stalder R., Mei J., Graham K. R., Estrada L. A., Reynolds J. R. (2014). Chem. Mater..

[cit58] Wang Y., Nakano M., Michinobu T., Kiyota Y., Mori T., Takimiya K. (2017). Macromolecules.

[cit59] Nakano M., Osaka I., Takimiya K. (2015). Macromolecules.

[cit60] Dai Y.-Z., Ai N., Lu Y., Zheng Y.-Q., Dou J.-H., Shi K., Lei T., Wang J.-Y., Pei J. (2016). Chem. Sci..

[cit61] Hwang Y.-J., Ren G., Murari N. M., Jenekhe S. A. (2012). Macromolecules.

[cit62] Zhao Z., Yin Z., Chen H., Zheng L., Zhu C., Zhang L., Tan S., Wang H., Guo Y., Tang Q., Liu Y. (2017). Adv. Mater..

[cit63] Chen H., Moser M., Wang S., Jellett C., Thorley K., Harrison G. T., Jiao X., Xiao M., Purushothaman B., Alsufyani M., Bristow H., De Wolf S., Gasparini N., Wadsworth A., McNeill C. R., Sirringhaus H., Fabiano S., McCulloch I. (2021). J. Am. Chem. Soc..

[cit64] Yue W., Nikolka M., Xiao M., Sadhanala A., Nielsen C. B., White A. J. P., Chen H. Y., Onwubiko A., Sirringhaus H., McCulloch I. (2016). J. Mater. Chem. C.

[cit65] Kim G., Han A.-R., Lee H. R., Lee J., Oh J. H., Yang C. (2014). Chem. Commun..

[cit66] Shi K., Zhang F., Di C. A., Yan T. W., Zou Y., Zhou X., Zhu D., Wang J. Y., Pei J. (2015). J. Am. Chem. Soc..

[cit67] Han J., Fan H., Zhang Q., Hu Q., Russell T. P., Katz H. E. (2021). Adv. Funct. Mater..

[cit68] Xiao M., Kang B., Lee S. B., Perdigão L. M. A., Luci A., Warr D. A., Senanayak S. P., Nikolka M., Statz M., Wu Y., Sadhanala A., Schott S., Carey R., Wang Q., Lee M., Kim C., Onwubiko A., Jellett C., Liao H., Yue W., Cho K., Costantini G., McCulloch I., Sirringhaus H. (2020). Adv. Mater..

[cit69] Mei J., Bao Z. (2014). Chem. Mater..

[cit70] Liu J., Qiu L., Portale G., Koopmans M., ten Brink G., Hummelen J. C., Koster L. J. A. (2017). Adv. Mater..

[cit71] Hodsden T., Thorley K. J., Panidi J., Basu A., Marsh A. V., Dai H., White A. J. P., Wang C., Mitchell W., Glöcklhofer F., Anthopoulos T. D., Heeney M. (2020). Adv. Funct. Mater..

[cit72] Northrup J. E. (2007). Phys. Rev. B: Condens. Matter Mater. Phys..

[cit73] Kanimozhi C., Yaacobi-Gross N., Burnett E. K., Briseno A. L., Anthopoulos T. D., Salzner U., Patil S. (2014). Phys. Chem. Chem. Phys..

[cit74] Kang I. L., Yun H. J., Sung Chung D., Kwon S.-K., Kim Y.-H. (2013). J. Am. Chem. Soc..

[cit75] Schmode P., Savva A., Kahl R., Ohayon D., Meichsner F., Dolynchuk O., Thurn-Albrecht T., Inal S., Thelakkat M. (2020). ACS Appl. Mater. Interfaces.

[cit76] Yu H., Park K. H., Song I., Kim M.-J., Kim Y.-H., Oh J. H. (2015). J. Mater. Chem. C.

[cit77] Lei T., Wang J. Y., Pei J. (2014). Chem. Mater..

[cit78] Moia D., Giovannitti A., Szumska A. A., Maria I. P., Rezasoltani E., Sachs M., Schnurr M., Barnes P. R. F., McCulloch I., Nelson J. (2019). Energy Environ. Sci..

[cit79] Szweda R., Chendo C., Charles L., Baxter P. N. W., Lutz J. F. (2017). Chem. Commun..

[cit80] Giovannitti A., Sbircea D. T., Inal S., Nielsen C. B., Bandiello E., Hanifi D. A., Sessolo M., Malliaras G. G., McCulloch I., Rivnay J. (2016). Proc. Natl. Acad. Sci. U. S. A..

[cit81] Giovannitti A., Nielsen C. B., Sbircea D. T., Inal S., Donahue M., Niazi M. R., Hanifi D. A., Amassian A., Malliaras G. G., Rivnay J., McCulloch I. (2016). Nat. Commun..

[cit82] Nielsen C. B., Giovannitti A., Sbircea D. T., Bandiello E., Niazi M. R., Hanifi D. A., Sessolo M., Amassian A., Malliaras G. G., Rivnay J., McCulloch I. (2016). J. Am. Chem. Soc..

[cit83] Paterson A. F., Singh S., Fallon K. J., Hodsden T., Han Y., Schroeder B. C., Bronstein H., Heeney M., McCulloch I., Anthopoulos T. D. (2018). Adv. Mater..

[cit84] Zhao Z., Yin Z., Chen H., Guo Y., Tang Q., Liu Y. (2017). J. Mater. Chem. C.

[cit85] Zheng Y.-Q., Lei T., Dou J.-H., Xia X., Wang J.-Y., Liu C.-J., Pei J. (2016). Adv. Mater..

[cit86] Lei T., Dou J.-H., Cao X.-Y., Wang J.-Y., Pei J. (2013). Adv. Mater..

[cit87] Lei T., Dou J.-H., Cao X.-Y., Wang J.-Y., Pei J. (2013). J. Am. Chem. Soc..

[cit88] Chen H. Y., Nikolka M., Wadsworth A., Yue W., Onwubiko A., Xiao M., White A. J. P., Baran D., Sirringhaus H., McCulloch I. (2018). Macromolecules.

[cit89] Shi Y., Guo H., Qin M., Zhao J., Wang Y., Wang H., Wang Y., Facchetti A., Lu X., Guo X. (2018). Adv. Mater..

[cit90] Liu C., Xu Y., Noh Y. Y. (2015). Mater. Today.

[cit91] Zhou Y., Fuentes-Hernandez C., Shim J., Meyer J., Giordano A. J., Li H., Winget P., Papadopoulos T., Cheun H., Kim J., Fenoll M., Dindar A., Haske W., Najafabadi E., Khan T. M., Sojoudi H., Barlow S., Graham S., Bredas J.-L., Marder S. R., Kahn A., Kippelen B. (2012). Science.

[cit92] Sun B., Hong W., Aziz H., Li Y. (2015). Polym. Chem..

[cit93] Iqbal H. F., Holland E. K., Anthony J. E., Jurchescu O. D. (2020). Mater. Horiz..

[cit94] Yan H., Chen Z., Zheng Y., Newman C., Quinn J. R., Dötz F., Kastler M., Facchetti A. (2009). Nature.

[cit95] Bucella S. G., Luzio A., Gann E., Thomsen L., McNeill C. R., Pace G., Perinot A., Chen Z., Facchetti A., Caironi M. (2015). Nat. Commun..

[cit96] Lee W., Lee C., Yu H., Kim D.-J., Wang C., Woo H. Y., Oh J. H., Kim B. J. (2016). Adv. Funct. Mater..

[cit97] Kim J.-S., Kim J.-H., Lee W., Yu H., Kim H. J., Song I., Shin M., Oh J. H., Jeong U., Kim T.-S., Kim B. J. (2015). Macromolecules.

[cit98] Kohn P., Huettner S., Komber H., Senkovskyy V., Tkachov R., Kiriy A., Friend R. H., Steiner U., Huck W. T. S., Sommer J.-U., Sommer M. (2012). J. Am. Chem. Soc..

[cit99] Planells M., Schroeder B. C., McCulloch I. (2014). Macromolecules.

[cit100] Chen H. Y., Yeh S. C., Chen C. T., Chen C. T. (2012). J. Mater. Chem..

[cit101] Kim Y., Long D. X., Lee J., Kim G., Shin T. J., Nam K.-W., Noh Y.-Y., Yang C. (2015). Macromolecules.

[cit102] Huang H., Chen Z., Ortiz R. P., Newman C., Usta H., Lou S., Youn J., Noh Y. Y., Baeg K. J., Chen L. X., Facchetti A., Marks T. (2012). J. Am. Chem. Soc..

[cit103] Hwang Y. J., Murari N. M., Jenekhe S. A. (2013). Polym. Chem..

[cit104] Wang Y., Hasegawa T., Matsumoto H., Mori T., Michinobu T. (2018). Adv. Mater..

[cit105] Wang Y., Hasegawa T., Matsumoto H., Michinobu T. (2019). J. Am. Chem. Soc..

[cit106] Fukutomi Y., Nakano M., Hu J.-Y., Osaka I., Takimiya K. (2013). J. Am. Chem. Soc..

[cit107] Wang Y., Takimiya K. (2020). Adv. Mater..

[cit108] Zhou E., Nakano M., Izawa S., Cong J., Osaka I., Takimiya K., Tajima K. (2014). ACS Macro Lett..

[cit109] Takimiya K., Nakano M. (2018). Bull. Chem. Soc. Jpn..

[cit110] Nakano M., Osaka I., Takimiya K. (2017). Adv. Mater..

[cit111] Lei T., Dou J.-H., Pei J. (2012). Adv. Mater..

[cit112] Rowland R. S., Taylor R. (1996). J. Phys. Chem..

[cit113] von Eller-Pandraud H. (1960). Acta Crystallogr..

[cit114] Bronstein H., Nielsen C. B., Schroeder B. C., McCulloch I. (2020). Nat. Rev. Chem..

[cit115] Gao Y., Deng Y., Tian H., Zhang J., Yan D., Geng Y., Wang F. (2017). Adv. Mater..

[cit116] Sui Y., Deng Y., Han Y., Zhang J., Hu W., Geng Y. (2018). J. Mater. Chem. C.

[cit117] Zhao X., Wen Y., Ren L., Ma L., Liu Y., Zhan X. (2012). J. Polym. Sci., Part A: Polym. Chem..

[cit118] Li Y., Singh S. P., Sonar P. (2010). Adv. Mater..

[cit119] Zhang Y., Tang L., Sun H., Ling S., Yang K., Uddin M. A., Guo H., Tang Y., Wang Y., Feng K., Shi Y., Liu J., Zhang S., Woo H. Y., Guo X. (2019). Macromol. Rapid Commun..

[cit120] Qiu G., Jiang Z., Ni Z., Wang H., Dong H., Zhang J., Zhang X., Shu Z., Lu K., Zhen Y., Wei Z., Hu W. (2017). J. Mater. Chem. C.

[cit121] Guo K., Bai J., Jiang Y., Wang Z., Sui Y., Deng Y., Han Y., Tian H., Geng Y. (2018). Adv. Funct. Mater..

[cit122] Yap B. K., Xia R., Campoy-Quiles M., Stavrinou P. N., Bradley D. D. C. (2008). Nat. Mater..

[cit123] Kline R. J., McGehee M. D., Kadnikova E. N., Liu J., Fréchet J. M. J. (2003). Adv. Mater..

[cit124] Wang Y., Guo H., Harbuzaru A., Uddin M. A., Arrechea-Marcos I., Ling S., Yu J., Tang Y., Sun H., López Navarrete J. T., Ortiz R. P., Woo H. Y., Guo X. (2018). J. Am. Chem. Soc..

[cit125] Feng K., Guo H., Wang J., Shi Y., Wu Z., Su M., Zhang X., Son J. H., Woo H. Y., Guo X. (2021). J. Am. Chem. Soc..

[cit126] Surgailis J., Savva A., Druet V., Paulsen B. D., Wu R., Hamidi-Sakr A., Ohayon D., Nikiforidis G., Chen X., McCulloch I., Rivnay J., Inal S. (2021). Adv. Funct. Mater..

[cit127] Briseno A. L., Kim F. S., Babel A., Xia Y., Jenekhe S. A. (2011). J. Mater. Chem..

[cit128] Guo X., Ponce Ortiz R., Zheng Y., Hu Y., Noh Y.-Y., Baeg K.-J., Facchetti A., Marks T. J. (2011). J. Am. Chem. Soc..

[cit129] Choi J., Song H., Kim N., Kim F. S. (2015). Semicond. Sci. Technol..

[cit130] Izuhara D., Swager T. M. (2009). J. Am. Chem. Soc..

[cit131] Hahm S. G., Rho Y., Jung J., Kim S. H., Sajoto T., Kim F. S., Barlow S., Park C. E., Jenekhe S. A., Marder S. R., Ree M. (2013). Adv. Funct. Mater..

[cit132] Guo K., Wu B., Jiang Y., Wang Z., Liang Z., Li Y., Deng Y., Geng Y. (2019). J. Mater. Chem. C.

[cit133] Pappa A.-M., Parlak O., Scheiblin G., Mailley P., Salleo A., Owens R. M. (2018). Trends Biotechnol..

[cit134] Gifford R. (2013). ChemPhysChem.

[cit135] Bernards D. A., Malliaras G. G. (2007). Adv. Funct. Mater..

[cit136] Friedlein J. T., McLeod R. R., Rivnay J. (2018). Org. Electron..

[cit137] Inal S., Malliaras G. G., Rivnay J. (2017). Nat. Commun..

[cit138] Giovannitti A., Nielsen C. B., Sbircea D. T., Inal S., Donahue M., Niazi M. R., Hanifi D. A., Amassian A., Malliaras G. G., Rivnay J., McCulloch I. (2016). Nat. Commun..

[cit139] Moser M., Hidalgo T. C., Surgailis J., Gladisch J., Ghosh S., Sheelamanthula R., Thiburce Q., Giovannitti A., Salleo A., Gasparini N., Wadsworth A., Zozoulenko I., Berggren M., Stavrinidou E., Inal S., McCulloch I. (2020). Adv. Mater..

[cit140] Donahue M. J., Williamson A., Strakosas X., Friedlein J. T., McLeod R. R., Gleskova H., Malliaras G. G. (2018). Adv. Mater..

[cit141] Flagg L. Q., Bischak C. G., Onorato J. W., Rashid R. B., Luscombe C. K., Ginger D. S. (2019). J. Am. Chem. Soc..

[cit142] Giovannitti A., Maria I. P., Hanifi D., Donahue M. J., Bryant D., Barth K. J., Makdah B. E., Savva A., Moia D., Zetek M., Barnes P. R. F., Reid O. G., Inal S., Rumbles G., Malliaras G. G., Nelson J., Rivnay J., McCulloch I. (2018). Chem. Mater..

[cit143] Savva A., Cendra C., Giugni A., Torre B., Surgailis J., Ohayon D., Giovannitti A., McCulloch I., Di Fabrizio E., Salleo A., Rivnay J., Inal S. (2019). Chem. Mater..

[cit144] Moser M., Ponder J. F., Wadsworth A., Giovannitti A., McCulloch I. (2019). Adv. Funct. Mater..

[cit145] Pappa A. M., Ohayon D., Giovannitti A., Maria I. P., Savva A., Uguz I., Rivnay J., McCulloch I., Owens R. M., Inal S. (2018). Sci. Adv..

[cit146] Ohayon D., Nikiforidis G., Savva A., Giugni A., Wustoni S., Palanisamy T., Chen X., Maria I. P., Di Fabrizio E., Costa P. M. F. J., Mcculloch I., Inal S. (2019). Nat. Mater..

[cit147] Wang C., Dong H., Hu W., Liu Y., Zhu D. (2011). Chem. Rev..

[cit148] Kim F. S., Guo X., Watson M. D., Jenekhe S. A. (2010). Adv. Mater..

[cit149] Chen X., Marks A., Paulsen B. D., Wu R., Rashid R. B., Chen H., Alsufyani M., Rivnay J., McCulloch I. (2021). Angew. Chem., Int. Ed..

[cit150] Kawan M., Hidalgo T. C., Du W., Pappa A.-M., Owens R. M., McCulloch I., Inal S. (2020). Mater. Horiz..

[cit151] Maria I. P., Paulsen B. D., Savva A., Ohayon D., Wu R., Hallani R., Basu A., Du W., Anthopoulos T. D., Inal S., Rivnay J., McCulloch I., Giovannitti A. (2021). Adv. Funct. Mater..

[cit152] Ohayon D., Savva A., Du W., Paulsen B. D., Uguz I., Ashraf R. S., Rivnay J., McCulloch I., Inal S. (2021). ACS Appl. Mater. Interfaces.

[cit153] Ohayon D., Inal S. (2020). Adv. Mater..

[cit154] Paterson A. F., Savva A., Wustoni S., Tsetseris L., Paulsen B. D., Faber H., Emwas A. H., Chen X., Nikiforidis G., Hidalgo T. C., Moser M., Maria I. P., Rivnay J., McCulloch I., Anthopoulos T. D., Inal S. (2020). Nat. Commun..

[cit155] Savva A., Hallani R., Cendra C., Surgailis J., Hidalgo T. C., Wustoni S., Sheelamanthula R., Chen X., Kirkus M., Giovannitti A., Salleo A., McCulloch I., Inal S. (2020). Adv. Funct. Mater..

[cit156] Savva A., Ohayon D., Surgailis J., Paterson A. F., Hidalgo T. C., Chen X., Maria I. P., Paulsen B. D., Petty A. J., Rivnay J., McCulloch I., Inal S. (2019). Adv. Electron. Mater..

[cit157] Arnold F. E., Van Deusen R. L. (1969). Macromolecules.

[cit158] Jenekhe S. A., Yi S. (2000). Appl. Phys. Lett..

[cit159] Wang S., Sun H., Ail U., Vagin M., Persson P. O. Å., Andreasen J. W., Thiel W., Berggren M., Crispin X., Fazzi D., Fabiano S. (2016). Adv. Mater..

[cit160] Kim F. S., Park C. H., Na Y., Jenekhe S. A. (2019). Org. Electron..

[cit161] Babel A., Jenekhe S. A. (2002). Adv. Mater..

[cit162] Jenekhe S. A., Tibbetts S. J. (1988). J. Polym. Sci., Part B: Polym. Phys..

[cit163] Lindorf M., Mazzio K. A., Pflaum J., Nielsch K., Brütting W., Albrecht M. (2020). J. Mater. Chem. A.

[cit164] Bubnova O., Crispin X. (2012). Energy Environ. Sci..

[cit165] Snyder G. J., Snyder A. H. (2017). Energy Environ. Sci..

[cit166] Zebarjadi M., Esfarjani K., Dresselhaus M. S., Ren Z. F., Chen G. (2012). Energy Environ. Sci..

[cit167] Lu Y., Yu Z.-D., Liu Y., Ding Y.-F., Yang C.-Y., Yao Z.-F., Wang Z.-Y., You H.-Y., Cheng X.-F., Tang B., Wang J.-Y., Pei J. (2020). J. Am. Chem. Soc..

[cit168] Lu Y., Wang J. Y., Pei J. (2019). Chem. Mater..

[cit169] Thomas E. M., Popere B. C., Fang H., Chabinyc M. L., Segalman R. A. (2018). Chem. Mater..

[cit170] Yang C.-Y., Ding Y.-F., Huang D., Wang J., Yao Z.-F., Huang C.-X., Lu Y., Un H.-I., Zhuang F.-D., Dou J.-H., Di C., Zhu D., Wang J.-Y., Lei T., Pei J. (2020). Nat. Commun..

[cit171] Wang S., Sun H., Erdmann T., Wang G., Fazzi D., Lappan U., Puttisong Y., Chen Z., Berggren M., Crispin X., Kiriy A., Voit B., Marks T. J., Fabiano S., Facchetti A. (2018). Adv. Mater..

[cit172] Wei P., Oh J. H., Dong G., Bao Z. (2010). J. Am. Chem. Soc..

[cit173] Zeng Y., Zheng W., Guo Y., Han G., Yi Y. (2020). J. Mater. Chem. A.

[cit174] Kiefer D., Giovannitti A., Sun H., Biskup T., Hofmann A., Koopmans M., Cendra C., Weber S., Anton Koster L. J., Olsson E., Rivnay J., Fabiano S., McCulloch I., Müller C. (2018). ACS Energy Lett..

[cit175] Guo S., Kim S. B., Mohapatra S. K., Qi Y., Sajoto T., Kahn A., Marder S. R., Barlow S. (2012). Adv. Mater..

[cit176] Lin X., Wegner B., Lee K. M., Fusella M. A., Zhang F., Moudgil K., Rand B. P., Barlow S., Marder S. R., Koch N., Kahn A. (2017). Nat. Mater..

[cit177] Liu J., Garman M. P., Dong J., Van Der Zee B., Qiu L., Portale G., Hummelen J. C., Koster L. J. A. (2019). ACS Appl. Energy Mater..

[cit178] Wang S., Ruoko T.-P., Wang G., Riera-Galindo S., Hultmark S., Puttisong Y., Moro F., Yan H., Chen W. M., Berggren M., Müller C., Fabiano S. (2020). ACS Appl. Mater. Interfaces.

[cit179] Boyle C. J., Upadhyaya M., Wang P., Renna L. A., Lu-Díaz M., Pyo Jeong S., Hight-Huf N., Korugic-Karasz L., Barnes M. D., Aksamija Z., Venkataraman D. (2019). Nat. Commun..

[cit180] Un H., Gregory S. A., Mohapatra S. K., Xiong M., Longhi E., Lu Y., Rigin S., Jhulki S., Yang C., Timofeeva T. V., Wang J., Yee S. K., Barlow S., Marder S. R., Pei J. (2019). Adv. Energy Mater..

[cit181] Liu J., Qiu L., Alessandri R., Qiu X., Portale G., Dong J., Talsma W., Ye G., Sengrian A. A., Souza P. C. T., Loi M. A., Chiechi R. C., Marrink S. J., Hummelen J. C., Koster L. J. A. (2018). Adv. Mater..

[cit182] Yan X., Xiong M., Li J. T., Zhang S., Ahmad Z., Lu Y., Wang Z. Y., Yao Z. F., Wang J. Y., Gu X., Lei T. (2019). J. Am. Chem. Soc..

[cit183] Bin Z., Liu Z., Qiu Y., Duan L. (2018). Adv. Opt. Mater..

[cit184] Liu J., Shi Y., Dong J., Nugraha M. I., Qiu X., Su M., Chiechi R. C., Baran D., Portale G., Guo X., Koster L. J. A. (2019). ACS Energy Lett..

[cit185] Lu Y., Yu Z., Zhang R., Yao Z., You H., Jiang L., Un H., Dong B., Xiong M., Wang J., Pei J. (2019). Angew. Chem., Int. Ed..

[cit186] Yang C.-Y., Jin W.-L., Wang J., Ding Y.-F., Nong S., Shi K., Lu Y., Dai Y.-Z., Zhuang F.-D., Lei T., Di C.-A., Zhu D., Wang J.-Y., Pei J. (2018). Adv. Mater..

[cit187] Schlitz R. A., Brunetti F. G., Glaudell A. M., Miller P. L., Brady M. A., Takacs C. J., Hawker C. J., Chabinyc M. L. (2014). Adv. Mater..

[cit188] Meng B., Liu J., Wang L. (2020). Polym. Chem..

[cit189] Liu J., Ye G., van der Zee B., Dong J., Qiu X., Liu Y., Portale G., Chiechi R. C., Koster L. J. A. (2018). Adv. Mater..

[cit190] Liu J., Ye G., Potgieser H. G. O., Koopmans M., Sami S., Nugraha M. I., Villalva D. R., Sun H., Dong J., Yang X., Qiu X., Yao C., Portale G., Fabiano S., Anthopoulos T. D., Baran D., Havenith R. W. A., Chiechi R. C., Koster L. J. A. (2021). Adv. Mater..

[cit191] Ye G., Liu J., Qiu X., Stäter S., Qiu L., Liu Y., Yang X., Hildner R., Koster L. J. A., Chiechi R. C. (2021). Macromolecules.

[cit192] Zhao X., Madan D., Cheng Y., Zhou J., Li H., Thon S. M., Bragg A. E., DeCoster M. E., Hopkins P. E., Katz H. E. (2017). Adv. Mater..

[cit193] Goel M., Heinrich C. D., Krauss G., Thelakkat M. (2019). Macromol. Rapid Commun..

[cit194] Meng B., Liu J., Wang L. (2020). Nano Mater. Sci..

[cit195] Wang H., Hsu J.-H., Yi S.-I., Kim S. L., Choi K., Yang G., Yu C. (2015). Adv. Mater..

[cit196] Lu Y., Yu Z. Z.-D., Zhang R. R.-Z., Yao Z. Z.-F., You H.-Y. H., Jiang L., Un H.-I. H., Dong B. B.-W., Xiong M., Wang J.-Y. J., Pei J. (2019). Angew. Chem., Int. Ed..

[cit197] Kim G., Han A.-R., Lee H. R., Lee J., Oh J. H., Yang C. (2014). Chem. Commun..

[cit198] Naab B. D., Gu X., Kurosawa T., To J. W. F., Salleo A., Bao Z. (2016). Adv. Electron. Mater..

